# Simple and Green Preparation of Tetraalkoxydiborons and Diboron Diolates from Tetrahydroxydiboron

**DOI:** 10.1021/acs.joc.3c02992

**Published:** 2024-04-19

**Authors:** Ryan M. Fornwald, Anshu Yadav, Jose R. Montero Bastidas, Milton R. Smith, Robert E. Maleczka

**Affiliations:** Department of Chemistry, Michigan State University, 578 South Shaw Lane, East Lansing, Michigan 48824, United States

## Abstract

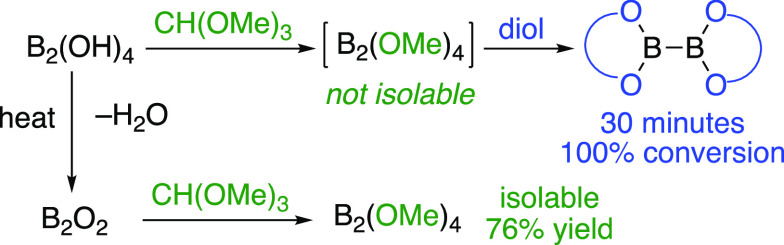

Tetraalkoxydiborons can be easily prepared by acid-catalyzed reactions of tetrahydroxydiboron or its anhydride with trialkyl orthoformates. Addition of diols to these reaction mixtures afforded diboron diolates in high yield. In both cases, removal of volatile byproducts is all that is required for the isolation of the diboron. These methods constitute a convenient alternative to previous preparations from tetrakis (dimethylamino) diboron and tetrahydroxydiboron.

## 1 Introduction

**Scheme 1:**
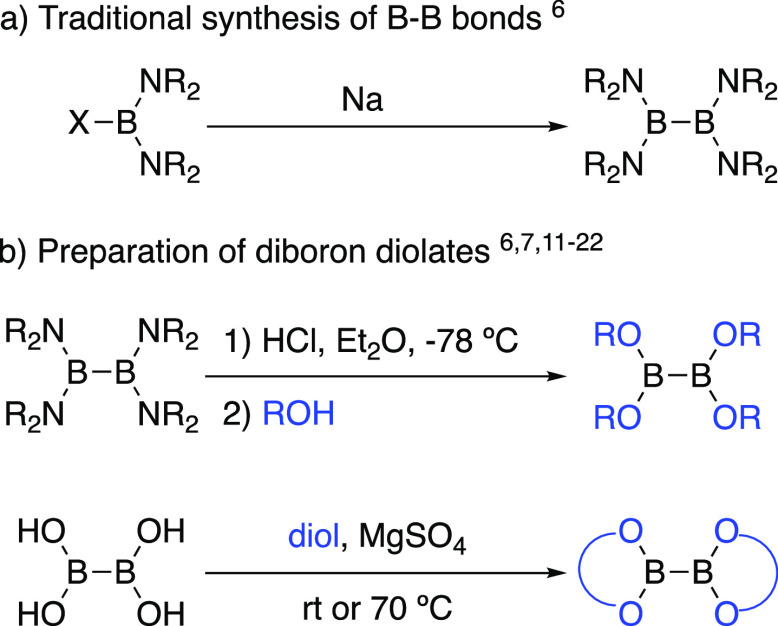
Previous Work

Diboron diolates have broad applications in synthesis,^1−5^ and while they are generally bench-stable compounds, their preparation can be tedious. Boron–boron bonds are commercially prepared via the Wurtz coupling of halobis (dialkylamino) boranes (Scheme 1a).^6,7^ While tetrakis (dialkylamino) diborons also have applications in synthesis,^8−10^ diboron diolates are far more widely used.^1−5^ Their direct preparation from tetrakis (dialkylamino) diborons requires the use of a dry solution of HCl in Et_2_O at −78 °C, filtration of dialkylammonium hydrochloride salts, then distillation, sublimation, or crystallization of the diboron diolate.^6,7^ However, B_2_(OH)_4_ (**1**) is also readily prepared via hydrolysis of tetrakis (dialkylamino)diboron.^11^ Diboron diolates can also be synthesized by stirring a heterogeneous mixture of B_2_(OH)_4_ with a diol and MgSO_4_ for 24 h (Scheme 1b).^12−22^

## 2 Results and Discussion

**Scheme 2:**
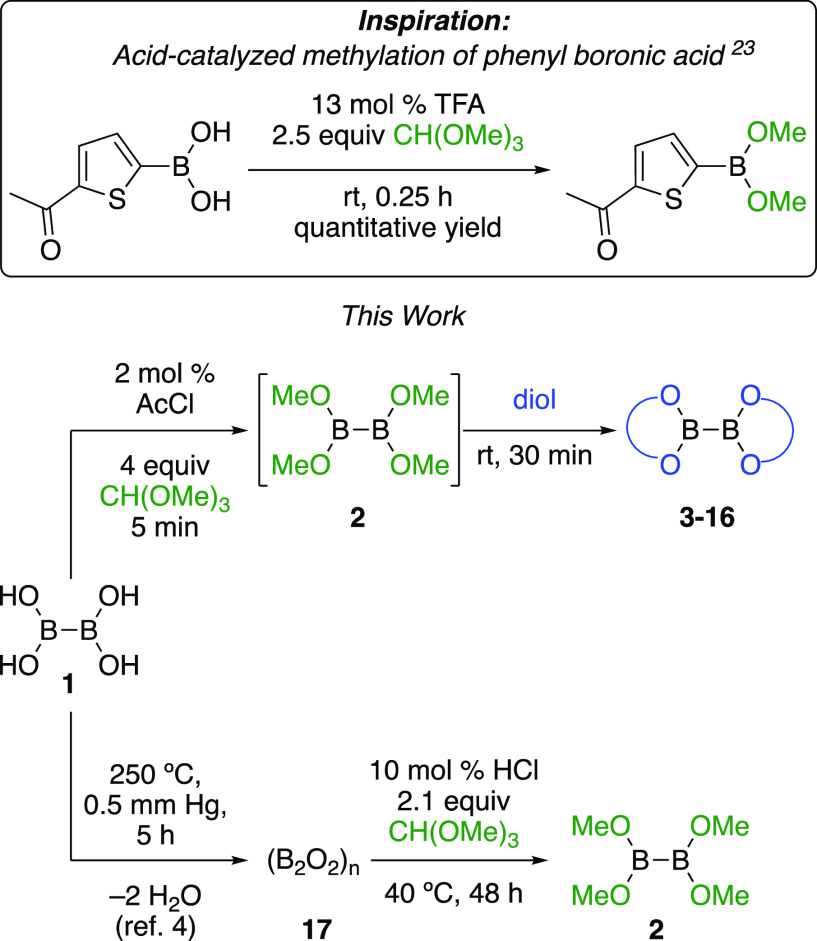
This Work

Inspired by a previous report for the preparation of thiophene boronic ester from thiophene boronic acid by reaction with trimethyl orthoformate in the presence of an acid catalyst,^23^ we have developed an exceptionally fast and convenient method for the preparation of diboron diolates and tetraalkoxydiborons (Scheme 2).

The acetyl chloride-catalyzed reaction of B_2_(OH)_4_ (**1**) with CH(OMe)_3_ is complete within minutes at room temperature, is not particularly air-sensitive, and offers a visual indicator of completion, producing a homogeneous solution (B_2_(OMe)_4_ (**2**), MeOH, and methyl formate) after consumption of starting material. The diol is then added, the solution stirred briefly, and solvent removed in vacuo to afford the desired diboron in high yield, typically without a need for further purification.

**Scheme 3:**
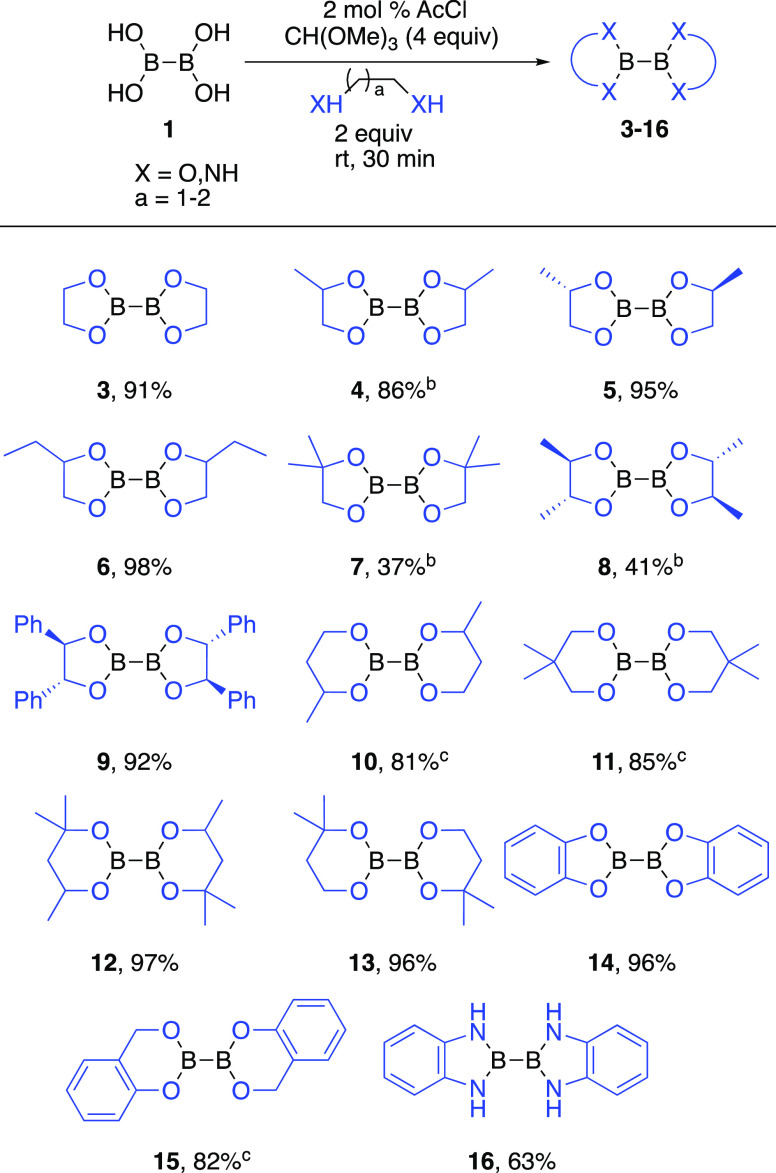
Reaction Scope Isolated yield. Distilled under vacuum. Isolated by DCM/water extraction.

A variety of diboron diolates (**3**–**16**) were prepared on a gram scale (Scheme 3) by using this methodology. Reactions with aliphatic 1,2-diols (**3**–**9**), aliphatic 1,3-diols (**10**–**13**), and phenols such as catechol or 2-hydroxybenzyl alcohol (**14**–**15**) were successful as well as 1,2-diaminobenzene (**16**) formed the desired product. Conversion was low with diisopropyl tartrate, and perfluoropinacol failed to afford any product. Attempts to separate B_2_(OMe)_4_ (**2**) from the MeOH byproduct by distillation were unsuccessful.

**Figure 1:**
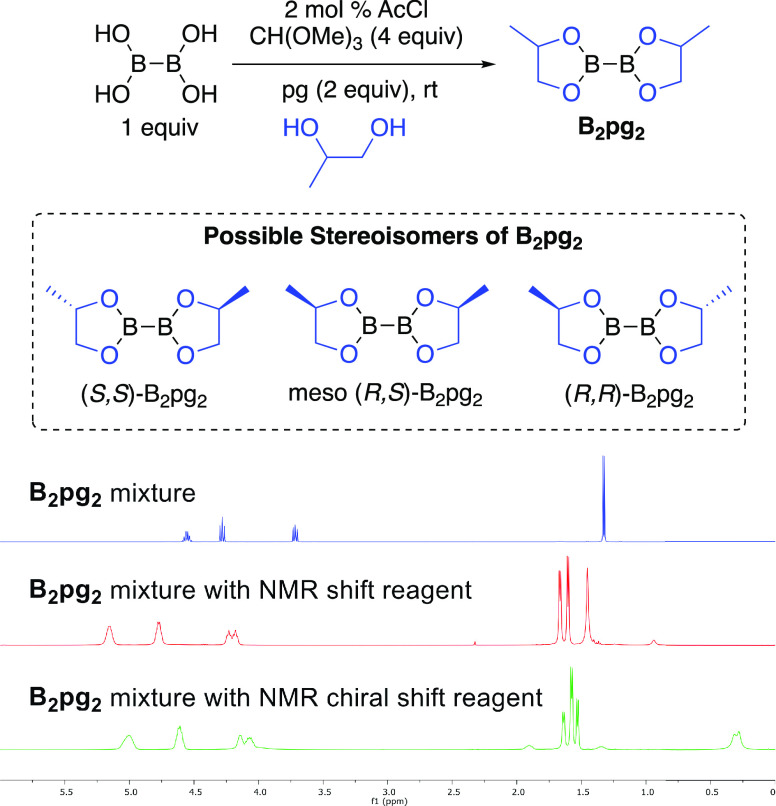
^1^H NMR spectra of a mixture of B_2_pg_2_ with NMR shift reagent Eu(fod)_3_ and optically active NMR chiral shift reagent Eu(hfc)_3_.

B_2_eg_2_ (**3**) and commonly used B_2_pin_2_ appear as white solids at room temperature, whereas synthetic B_2_pg_2_ (**4**) is a colorless liquid. B_2_pg_2_ was made starting with a racemic mixture of propylene glycol that should lead to a mixture of diastereomers and explain its liquid phase (Figure 1). In fact, when the pure enantiomer (*S*)-propylene glycol is used as the starting diol, (*S,S*)-B_2_pg_2_ is obtained as a crystalline solid. To measure the ratio of the stereoisomers in the mixture of B_2_pg_2_ diastereomers, we added NMR shift reagents. The methyl groups in the B_2_pg_2_ mixture appear as one peak, which splits into different ones after the addition of the NMR shift reagent. As expected, the two B_2_pg_2_ diastereomers are present in a 50:50 mixture corresponding to the meso isomer (*S,R*)-B_2_pg_2_ and an equimolar mixture of the enantiomers (*S,S*)-B_2_pg_2_ and (*R,R*)-B_2_pg_2_.

**Scheme 4:**
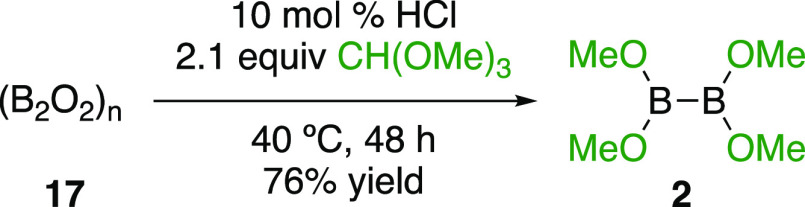
Synthesis of Tetramethoxydiboron

Interestingly, the anhydride of B_2_(OH)_4_ can be prepared quantitatively by heating under vacuum for several hours, generating (B_2_O_2_)_*n*_ (**17**) as a hard white solid (Scheme 2).^11,24^ This material failed to react with CH(OMe)_3_ in the presence of catalytic AcCl, but did so with dry HCl in Et_2_O (Scheme 4). These reactions are much slower than those with B_2_(OH)_4_ (**1**) and require gentle heating. Removal of residual (B_2_O_2_)_*n*_ by filtration, and Et_2_O and methyl formate by vacuum distillation with a water aspirator afforded B_2_(OMe)_4_ in good yield and 91% purity (containing 4% methyl formate, 2% MeOH, and 3% Et_2_O)_._ This is a simple alternative to previous methods for its synthesis from tetrakis(dialkylamino)diborons.^6^

We also attempted to prepare B_2_(OEt)_4_ via this method and obtained a mixture whose primary spectral features were consistent with its structure. However, our assignment is not secure due to the complexity of the spectra. (*Caution: unlike B*_*2*_*(OMe)*_*4*_*, the reaction of B*_*2*_*(OEt)*_*4*_*with air is spontaneous and very exothermic!*)

## 3 Conclusions

In conclusion, we have developed a simple, convenient, and high yielding method for the preparation of both diboron diolates and tetraalkoxydiborons using bench-stable B_2_(OH)_4_ as a universal precursor.

## 4 Experimental Section

### 4.1 General Information

All diols were purchased from Sigma except ethane-1,2-diol and 3-methylbutane-1,3-diol, which were purchased from Fischer Chemicals and Oakwood Chemical respectively. Benzene-1,2-diamine was purchased from Eastman Chemicals. All commercial chemicals were used as received unless otherwise indicated. ^1^H, ^13^C, and ^11^B NMR spectra were recorded on a Varian 500 MHz DD2 Spectrometer equipped with a ^1^H–^19^F/^15^N–^31^P 5 mm Pulsed Field Gradient (PFG) probe. Chemical shifts are reported as parts per million (ppm). Splitting patterns are designated as singlet (s), doublet (d), triplet (t), quartet (q), doublet of doublet (dd), and doublet of doublet of doublets (ddd). NMR spectra were processed for display using the MNova software program with only phasing and baseline corrections applied. High-resolution mass spectra (HRMS) were recorded on a Leco GC-ToF spectrometer. All reactions were carried out under a nitrogen atmosphere in oven-dried glassware. Anhydrous dichloromethane was obtained from commercial sources and was used without further purification. Boron monoxide ((B_2_O_2_)_*n*_) was prepared as previously described in the literature.^11,24^ The reported yields describe the results of a single experiment.

### 4.2 General Experimental Procedure

An oven-dried round-bottom flask was charged with B_2_(OH)_4_ (1.0 equiv) and CH(OMe)_3_ (4.0 equiv) and a stir bar. AcCl was added (0.02 equiv), the vessel was flushed with N_2_ and sealed with a silicone septum, and the mixture was stirred rapidly at room temperature until all solid material had dissolved (5 min or less, determined by stir rate and the grain size of the B_2_(OH)_4_). The diol was then added, and the mixture was stirred for 30 min. Solvent was removed in vacuo via rotary evaporation and then under high vacuum to afford the title compound.

#### 4.2.1 Tetramethoxydiboron (B_2_(OMe)_4_) (**2**)

A 1 dram vial was charged with a stir bar, (B_2_O_2_)_*n*_ (268 mg, 5 mmol), CH(OMe)_3_ (1.15 mL, 10.5 mmol), and 2.0 M HCl in Et_2_O (0.25 mL, 0.5 mmol). The vial was flushed with N_2_ and capped and then stirred at 40 °C in an oil bath for 48 h. The cap was replaced with a septum and connected to a water aspirator by a length of PTFE tubing. HCO_2_Me and Et_2_O were removed via vacuum distillation at room temperature, gradually raising the temperature over 1 h to 40 °C in an oil bath. The remaining liquid was then filtered through glass wool under a N_2_ atmosphere. This procedure afforded 608 mg of the title compound in 91% purity (containing 4% methyl formate, 2% methanol, 3% diethyl ether) as a colorless oil for an overall yield of 553 mg (76%). The relative proportions of each component were estimated via spectral deconvolution using the line fitting protocol in Mnova 14.2.0 (Mestrelab Research, S.L.; Santiago de Compostela, Spain) using Gaussian/Lorentzian peak shapes and simulated annealing. Spectral data are for the title compound. ^1^H NMR (500 MHz, CDCl_3_): δ 3.62 (s, 12H); ^13^C{^1^H} NMR (126 MHz, CDCl_3_): δ 52.1; ^11^B NMR (160 MHz, CDCl_3_): δ 31.2. (For NMR spectra in C_7_D_8_ see Braunschweig, H.; Damme, A. Thermodynamic control of oxidative addition and reductive elimination processes in *cis*-bis(dimethoxyboryl)-bis(tricyclohexylphosphine)platinum(II). *Chem. Commun.***2013**, *49* (45), 5216–5218).

#### 4.2.2 2,2′-Bi(1,3,2-dioxaborolane) (B_2_eg_2_) (**3**)

The title compound was prepared from B_2_(OH)_4_ (2.24 g, 25 mmol), CH(OMe)_3_ (10.94 mL, 100 mmol), AcCl (18 μL, 0.25 mmol), and ethylene glycol (2.8 mL, 50 mmol) according to the general procedure. This procedure afforded 3.22 g (91%) of the title compound as a white solid: mp = 163–164 °C (lit mp = 159–160 °C^16^). ^1^H and ^13^C, data were inconsistent with literature values as impurities were present in the literature spectrum.^16^^1^H NMR (500 MHz, CDCl_3_):^16^ δ 4.18 (s, 8H); ^13^C NMR (126 MHz, CDCl_3_):^16^ δ 65.7; ^11^B NMR (160 MHz, CDCl_3_):^16^ δ 30.82. GC-MS (EI) *m/*z calcd for C_4_H_8_B_2_O_4_ [M]^+^ 142.06, found: 142.1.

#### 4.2.3 4,4′-Dimethyl-2,2′-bi(1,3,2-dioxaborolane) (B_2_pg_2_) (**4**)

The title compound was prepared from B_2_(OH)_4_ (2.24 g, 25 mmol), CH(OMe)_3_ (10.94 mL, 100 mmol), AcCl (18 μL, 0.25 mmol), and propylene glycol (3.65 mL, 50 mmol) according to the general procedure. The mixture was concentrated and then distilled at 0.07 torr to afford 3.64 g (86%) of the title compound as a colorless oil. The title compound has been reported before, but no spectroscopic data were provided. ^1^H NMR (500 MHz, CDCl_3_): δ 4.57–4.50 (m, 2H), 4.26 (dd, *J* = 8.0, 0.9 Hz, 2H), 3.70 (ddd, *J* = 8.3, 7.4, 1.0 Hz, 2H), 1.31 (dd, *J* = 6.2, 0.5 Hz, 6H); ^13^C{^1^H} NMR (126 MHz, CDCl_3_): δ 73.6, 73.6, 72.1, 21.8; ^11^B NMR (160 MHz, CDCl_3_): δ 30.7. HRMS (GC/ToF) *m/*z calc for C_6_H_12_B_2_O_4_ [M]^+^ 170.0922, found: 170.0911.

#### 4.2.4 (4S,4′*S*) 4,4′-Dimethyl-2,2′-bi(1,3,2-dioxaborolane) ((S,S)-B_2_pg_2_) (**5**)

The title compound was prepared from B_2_(OH)_4_ (2.24 g, 25 mmol), CH(OMe)_3_ (10.94 mL, 100 mmol), AcCl (18 μL, 0.25 mmol), and (S)-propylene glycol (3.65 mL, 50 mmol) according to the general procedure. This procedure afforded 4.04 g (95%) of the title compound as a white solid: mp = 152–154 °C. ^1^H NMR (500 MHz, CDCl_3_): δ 4.63–4.46 (m, 2H), 4.24 (dd, *J* = 8.2, 1.0 Hz, 2H), 3.68 (dd, *J* = 7.4, 1.5 Hz, 2H), 1.29 (d, *J* = 6.3 Hz, 6H). ^13^C NMR (126 MHz, CDCl_3_): δ 73.5, 72.1, 21.8. ^11^B NMR (160 MHz, CDCl_3_): δ 30.66. HRMS (GC/ToF) *m/*z calc for C_6_H_12_B_2_O_4_ [M]^+^ 170.0922, found: 170.0664.

#### 4.2.5 4,4′-Diethyl-2,2′-bi(1,3,2-dioxaborolane) (B_2_bg_2_) (**6**)

The title compound was prepared from B_2_(OH)_4_ (2.24 g, 25 mmol), CH(OMe)_3_ (10.94 mL, 100 mmol), AcCl (18 μL, 0.25 mmol), and 1,2-butanediol (4.5 mL, 50 mmol) according to the general procedure. This procedure afforded 4.85 g (98%) of the title compound as a colorless oil. ^1^H NMR (500 MHz, CDCl_3_): δ 4.39–4.33 (m, 2H), 4.24 (dd, *J* = 8.3, 0.5 Hz, 2H), 3.78 (ddd, *J* = 8.9, 7.4, 1.7 Hz, 2H), 1.70–1.53 (m, 4H), 0.95 (t, *J* = 7.4 Hz, 6H). ^13^C{^1^H} NMR (126 MHz, CDCl_3_): δ 78.6, 70.4, 29.0, 29.0, 9.3. ^11^B NMR (160 MHz, CDCl_3_): δ 30.65. HRMS (GC/ToF) *m/*z calc for C_8_H_16_B_2_O_4_ [M]^+^ 198.1235, found: 198.1224.

#### 4.2.6 4,4,4′,4′-Tetramethyl-2,2′-bi(1,3,2-dioxaborolane) (**7**)

B_2_(OH)_4_ (896 mg, 10 mmol) and CH(OMe)_3_ (4.24 g, 40 mmol) were stirred in a Schlenk flask and the suspension was degassed with N_2_ for 15 min. One drop of acetyl chloride, AcCl, was added, and the mixture became a homogeneous solution. 2-methyl-1,2-propandiol (mpg, 1.80 g, 20 mmol) was added, and the reaction was stirred at room temperature overnight. The mixture was concentrated and then distilled under reduced pressure to yield 730 mg of the title compound as a colorless oil (37% yield). The ^1^H NMR spectrum is inconsistent with literature due to different field strengths. However, our ^1^H, ^13^C, and ^11^B are self-consistent.^25^^1^H NMR (500 MHz, CDCl_3_): δ 3.89 (s, 4H), 1.36 (s, 12H). ^13^C{^1^H} NMR (126 MHz, CDCl_3_): δ 80.5, 77.4, 28.6. ^11^B NMR (160 MHz, CDCl_3_): δ 30.80. GC-MS (EI) *m/*z calcd for C_8_H_16_B_2_O_4_ [M]^+^ 198.1, found: 198.1.

#### 4.2.7 (4*R*,4′*R*,5*R*,5′*R*)-4,4′,5,5′-Tetramethyl-2,2′-bi(1,3,2-dioxaborolane) (**8**)

B_2_(OH)_4_ (896 mg, 10 mmol) and CH(OMe)_3_ (4.24 g, 40 mmol) were stirred in a Schlenk flask, and the suspension was degassed with N_2_ for 15 min. One drop of acetyl chloride, AcCl, was added, and the mixture became a homogeneous solution. (2R,3R)-butanediol (1.80 g, 20 mmol) was added, and the reaction was stirred at room temperature overnight. The mixture was concentrated and then distilled under reduced pressure to yield 820 mg of the title compound as a colorless oil (41% yield) that matched previously reported spectra.^25^^1^H NMR (500 MHz, CDCl_3_): δ 3.99–3.93 (m, 4H), 1.28–1.23 (m, 12H). ^13^C{^1^H} NMR (126 MHz, CDCl_3_): δ 80.3, 20.9. ^11^B NMR (160 MHz, CDCl_3_): δ 30.5. GC-MS (EI) *m/*z calcd for C_8_H_16_B_2_O_4_ [M]^+^ 198.1, found: 198.1.

#### 4.2.8 (4*R*,4′*R*,5*R*,5′*R*)-4,4′,5,5′-Tetraphenyl-2,2′-bi(1,3,2-dioxaborolane) (**9**)

The title compound was prepared from B_2_(OH)_4_ (0.56 g, 6.25 mmol), CH(OMe)_3_ (2.73 mL, 25 mmol), 2 mol % AcCl (4.5 μL, 0.0625 mmol), and (1R,2R)-1,2-diphenylethylene glycol (2.67 g, 12.5 mmol) according to the general procedure. This procedure afforded 2.57 g (92%) of the title compound as a pale pink-white solid: mp = 191–194 °C. ^1^H and ^13^C NMR data were consistent with literature values.^26^^1^H NMR (500 MHz, CDCl_3_):^26^ δ 7.45–7.35 (m, 20H), 5.30 (s, 4H); ^13^C{^1^H} NMR (126 MHz, CDCl_3_):^26^ δ 139.9, 128.9, 128.5, 126.1, 86.8; ^11^B NMR (160 MHz, CDCl_3_):^26^ δ 31.46.

#### 4.2.9 4,4′-Dimethyl-2,2′-bi(1,3,2-dioxaborinane) (**10**)

The title compound was prepared from B_2_(OH)_4_ (1.12 g, 12.5 mmol), CH(OMe)_3_ (5.47 mL, 50 mmol), AcCl (9 μL, 0.125 mmol), and 1,3-butanediol (2.27 mL, 25 mmol) according to the general procedure. DCM/water extraction was performed to obtain a pure compound. This procedure afforded 2.0 g (81%) of the title compound as a viscous oil. ^1^H NMR spectrum is inconsistent with the literature due to different field strengths but our ^1^H, ^13^C, and ^11^B are self-consistent.^25^^1^H NMR (500 MHz, CDCl_3_):^25^ δ 4.13–4.06 (m, 2H), 4.02–3.98 (m, 2H), 3.95–3.90 (m, 2H), 1.94–1.88 (m, 2H), 1.73–1.66 (m, 2H), 1.28 (d, *J* = 6.3 Hz, 6H); ^13^C{^1^H} NMR (126 MHz, CDCl_3_):^25^ δ 66.8, 60.6, 60.5, 34.3, 34.3, 22.9, 22.9; ^11^B NMR (160 MHz, CDCl_3_):^25^ δ 28.04. GC-MS (EI) *m/*z calcd for C_8_H_16_B_2_O_4_ [M]^+^ 198.1, found: 198.1.

#### 4.2.10 5,5,5′,5′-Tetramethyl-2,2′-bi(1,3,2-dioxaborinane) (**11**)

The title compound was prepared from B_2_(OH)_4_ (1.12 g, 12.5 mmol), CH(OMe)_3_ (5.47 mL, 50 mmol), AcCl (9 μL, 0.125 mmol), and neopentyl glycol (2.6 g, 25 mmol) according to the general procedure. DCM/water extraction was performed to obtain a pure compound. This procedure afforded 2.4 g (85%) of the title compound as a white solid that matched previously reported spectra.^27^ mp = 184–186 °C (lit mp = 182.5–184.5)^28^^1^H NMR (500 MHz, CDCl_3_):^27^ δ 3.59 (s, 8H), 0.94 (s, 12H); ^13^C{^1^H} NMR (126 MHz, CDCl_3_):^27^ δ 71.6, 31.8, 22.2; ^11^B NMR (160 MHz, CDCl_3_):^27^ δ 27.76. GC-MS (EI) *m/*z calcd for C_10_H_20_B_2_O_4_ [M]^+^ 226.15, found: 226.1.

#### 4.2.11 4,4,4′,4′,6,6′-Hexamethyl-2,2′-bi(1,3,2-dioxaborinane) (**12**)

The title compound was prepared from B_2_(OH)_4_ (1.12 g, 12.5 mmol), CH(OMe)_3_ (5.47 mL, 50 mmol), AcCl (9 μL, 0.125 mmol), and hexylene glycol (3.21 mL, 25 mmol) according to the general procedure. This procedure afforded 3.0 g (97%) of the title compound as a white solid that matched previously reported spectra.^27^ mp = 102–104 °C (lit mp = 99–101 °C)^28^^1^H NMR (500 MHz, CDCl_3_):^27^ δ 4.21–4.10 (m, 2H), 1.75–1.71 (dt, *J* = 13.8, 2.8 Hz, 2H), 1.50 (dd, *J* = 11.6, 2.0 Hz, 2H), 1.28 (d, *J* = 4.2 Hz, 12H), 1.24 (dd, *J* = 6.2, 1.2 Hz, 6H); ^13^C{^1^H} NMR (126 MHz, CDCl_3_):^27^ δ 70.2, 70.1, 64.1, 64.1, 46.3, 46.3, 31.3, 31.2, 28.5, 28.4, 23.3, 23.2; ^11^B NMR (160 MHz, CDCl_3_):^27^ δ 28.01. GC-MS (EI) *m/*z calcd for C_12_H_24_B_2_O_4_ [M]^+^ 254.19, found: 254.15.

#### 4.2.12 4,4,4′,4′-Tetramethyl-2,2′-bi(1,3,2-dioxaborinane) (**13**)

The title compound was prepared from B_2_(OH)_4_ (2.24 g, 25.0 mmol), CH(OMe)_3_ (10.94 mL, 100 mmol), AcCl (18 μL, 0.25 mmol), and 3-methylbutane-1,3-diol (9.15 mL, 50 mmol) according to the general procedure. This procedure afforded 5.04 g (96%) of the title compound as a white solid that matched previously reported spectra.^25^ mp = 62–64 °C ^1^H NMR (500 MHz, CDCl_3_):^25^ δ 3.99 (m, 4H), 1.78 (m, 4H), 1.31 (s, 12H); ^13^C{^1^H} NMR (126 MHz, CDCl_3_):^25^ δ 69.6, 58.7, 38.5, 29.5; ^11^B NMR (160 MHz, CDCl_3_):^25^ δ 28.01. GC-MS (EI) *m/*z calcd for C_10_H_20_B_2_O_4_ [M]^+^ 226.15, found: 226.15.

#### 4.2.13 2,2′-Bibenzo[*d*][1,3,2]dioxaborole (**14**)

The title compound was prepared from B_2_(OH)_4_ (1, 1.12 g, 12.5 mmol), CH(OMe)_3_ (5.47 mL, 50 mmol), AcCl (9 μL, 0.125 mmol), and catechol (2.75 g, 25 mmol) according to the general procedure. This procedure afforded 2.85g (96%) of the title compound as an off-white solid that matched previously reported spectra.^29^ mp = 192–195 °C (lit mp = 195–198 °C)^30^^1^H NMR (500 MHz, CDCl_3_):^29^ δ 7.41–7.37 (m, 4H), 7.21–7.17 (m, 4H); ^13^C{^1^H} NMR (126 MHz, CDCl_3_):^29^ δ 147.8, 123.6, 113.2; ^11^B NMR (160 MHz, CDCl_3_):^29^ δ 30.76. GC-MS (EI) *m/*z calcd for C_12_H_8_B_2_O_4_ [M]^+^ 238.06, found: 238.06.

#### 4.2.14 4*H*,4′*H*-2,2′-Bibenzo[*d*][1,3,2]dioxaborinine (**15**)

The title compound was prepared from B_2_(OH)_4_ (1, 1.12 g, 12.5 mmol), CH(OMe)_3_ (5.47 mL, 50 mmol), AcCl (9 μL, 0.125 mmol), and 2-hydroxybenzyl alcohol (3.1 g, 25 mmol) according to the general procedure. DCM/water extraction was performed to obtain a pure compound. This procedure afforded 2.72 g (82%) of the title compound as an orange solid (mp = 160–164 °C) that matched previously reported data.^25^^1^H NMR (500 MHz, CDCl_3_):^25^ δ 7.23–7.19 (m, 2H), 7.07 (dd, *J* = 8.0, 0.9 Hz, 2H), 7.03 (td, *J* = 7.4, 1.2 Hz, 2H), 6.93 (dd, *J* = 7.5, 1.0 Hz, 2H), 5.12 (s, 4H); ^13^C{^1^H} NMR (126 MHz, CDCl_3_):^25^ δ 147.9, 128.9, 125.0, 123.6, 122.8, 118.2, 62.1; ^11^B NMR (160 MHz, CDCl_3_):^25^ δ 28.35. GC-MS (EI) *m/*z calcd for C_14_H_12_B_2_O_4_ [M]^+^ 266.09, found: 266.05.

#### 4.2.15 1,1′,3,3′-Tetrahydro-2,2′-bibenzo[*d*][1,3,2]diazaborole (**16**)

The title compound was prepared from B_2_(OH)_4_ (0.56 g, 6.25 mmol), CH(OMe)_3_ (2.73 mL, 25 mmol), AcCl (4.5 μL, 0.0625 mmol), and 1,2-diaminobenzene (1.35 g, 12.5 mmol) according to the general procedure. The crude product was dissolved into hot dry tetrahydrofuran (THF), filtered through a frit funnel, and transferred into a Schlenk tube, and the solvent was removed by vacuum. Redissolution in the THF followed by solvent diffusion of hexane from an overlayer at room temperature afforded 0.92 g (63%) of the title compound as an off-white solid that matched previously reported spectra.^31^ (mp = 316-319 °C) ^1^H NMR (500 MHz, (CD_3_)SO):^31^ δ 8.77 (s, 4H), 7.12–7.09 (m, 4H), 6.83–6.79 (m, 4H); ^13^C{^1^H} NMR (126 MHz, (CD_3_)SO):^31^ δ 137.3, 118.1, 110.8; ^11^B NMR (160 MHz, dmf-d_7_):^31^ δ 27.75. GC-MS (EI) *m/*z calcd for C_12_H_12_B_2_N_4_ [M]^+^ 234.1, found: 234.1.

#### 4.2.16 Procedure for Mixture of B_2_pg_2_ with NMR Shift Reagents

(a) In an oven-dried NMR tube, a mixture of NMR shift reagent Eu(fod)_3_ (11.2 mg, 0.01 mmol) and B_2_pg_2_ (11.9 mg, 0.07 mmol) was made in 1 mL of CDCl_3_. (b) In an oven-dried NMR tube, a mixture of optical active NMR chiral shift reagent Eu(hfc)_3_ (11.9 mg, 0.01 mmol) and B_2_pg_2_ (11.9 mg, 0.07 mmol) was made in 0.7 mL of CDCl_3_. The associated spectra were then acquired on a Varian 500 MHz DD2 NMR Spectrometer.

## Data Availability

The data underlying this study are available in the published article and its Supporting Information.

## Supporting Information Available

Experimental details and compound characterization data (PDF)
