# Supplementary material for: Simple and Green Preparation of Tetraalkoxydiborons and Diboron Diolates from Tetrahydroxydiboron
Source: J Org Chem. 2024 Apr 19;89(9):6048–52. doi: 10.1021/acs.joc.3c02992 (PMC11077490; doi:10.1021/acs.joc.3c02992)

Supporting Information

Simple and Green Preparation of Tetraalkoxydiborons and Diboron Diolates from Tetrahydroxydiboron

Ryan M. Fornwald†, Anshu Yadav†, Jose R. Montero Bastidas, Milton R. Smith, III,* Robert E. Maleczka Jr.,*

Department of Chemistry, Michigan State University, East Lansing, Michigan 48824-1322, United States

Robert E. Maleczka Jr. – maleczka@chemistry.msu.edu
Milton R. Smith III – smithmil@msu.edu

Table of Contents

TABLE OF CONTENTS S1

GENERAL INFORMATION S2

NMR SPECTRUM..S3

General Information

All diols were purchased from Sigma except ethane-1,2-diol and 3-methylbutane-1,3-diol, which were purchased from Fischer Chemicals and Oakwood Chemical respectively. Benzene-1,2-diamine was purchased from Eastman chemicals. All commercially available chemicals were used as received unless otherwise indicated. 1H, 13C and 11B NMR spectra were recorded on a varian 500 MHz DD2 Spectrometer equipped with a 1H-19F/15N-31P 5 mm Pulsed Field Gradient (PFG) probe. Chemical shifts are reported as hertz (Hz). Splitting patterns are designated as singlet (s), doublet (d), triplet (t), quartet (q), doublet of doublet (dd), doublet of doublet of doublets (ddd). NMR spectra were processed for display using the MNova software program with only phasing and baseline corrections applied. High resolution mass spectra (HRMS) were recorded on a Leco GC-ToF spectrometer. All reactions were carried out under a nitrogen atmosphere in oven-dried glassware. Anhydrous dichloromethane was obtained from commercial sources and used without further purification. Boron monoxide ($(B_2O_2)_n$) was prepared as previously described in the literature. The yields reported describe the result of a single experiment.

NMR SPECTRUM

^{1}H NMR of *Tetramethoxydiboron* (2) ($CDCl_3$, 500 MHz)

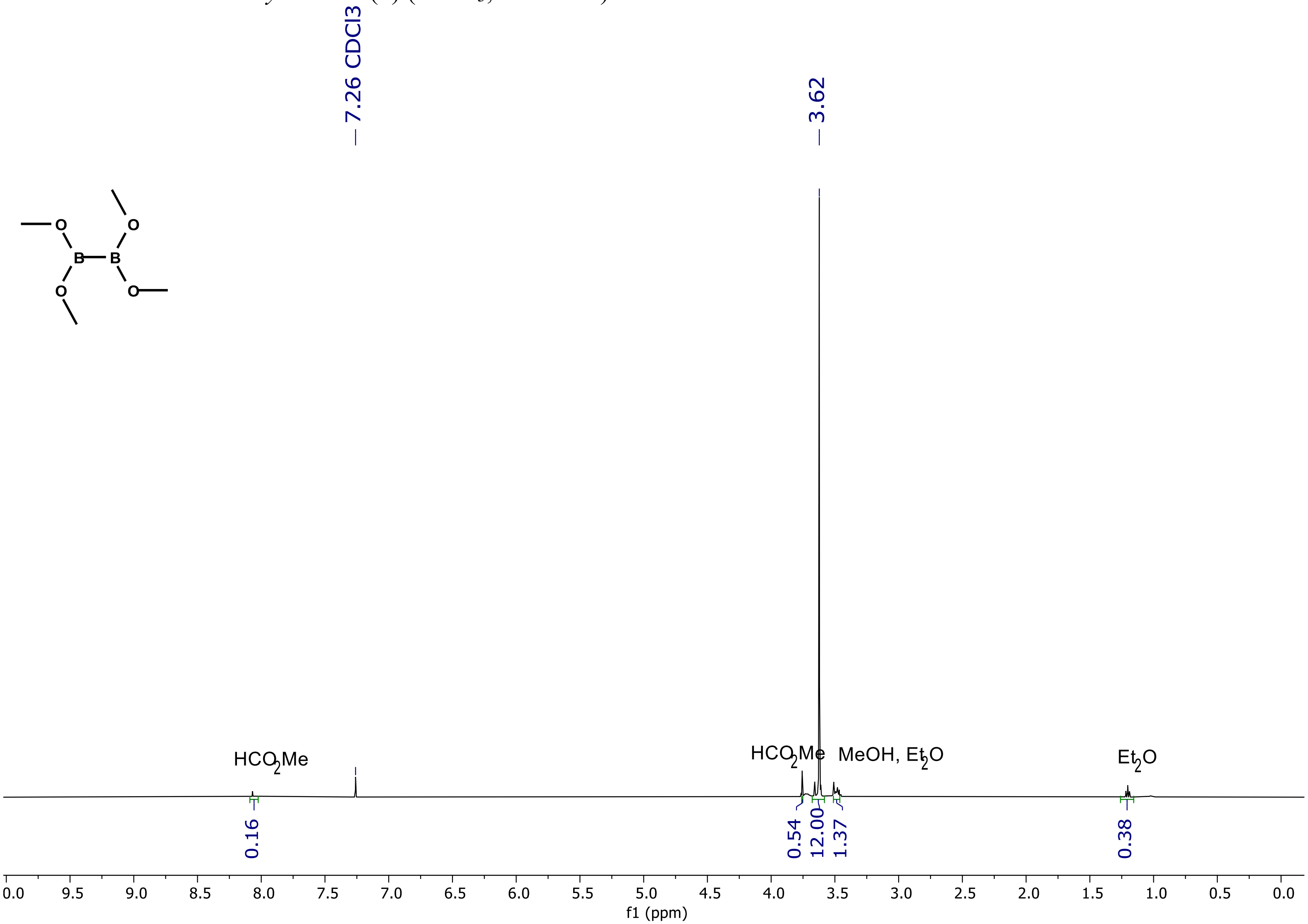

$^{13}C\{^1H\}$ NMR of *Tetramethoxydiboron* (2) ($CDCl_3$, 126 MHz)

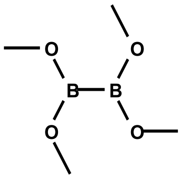

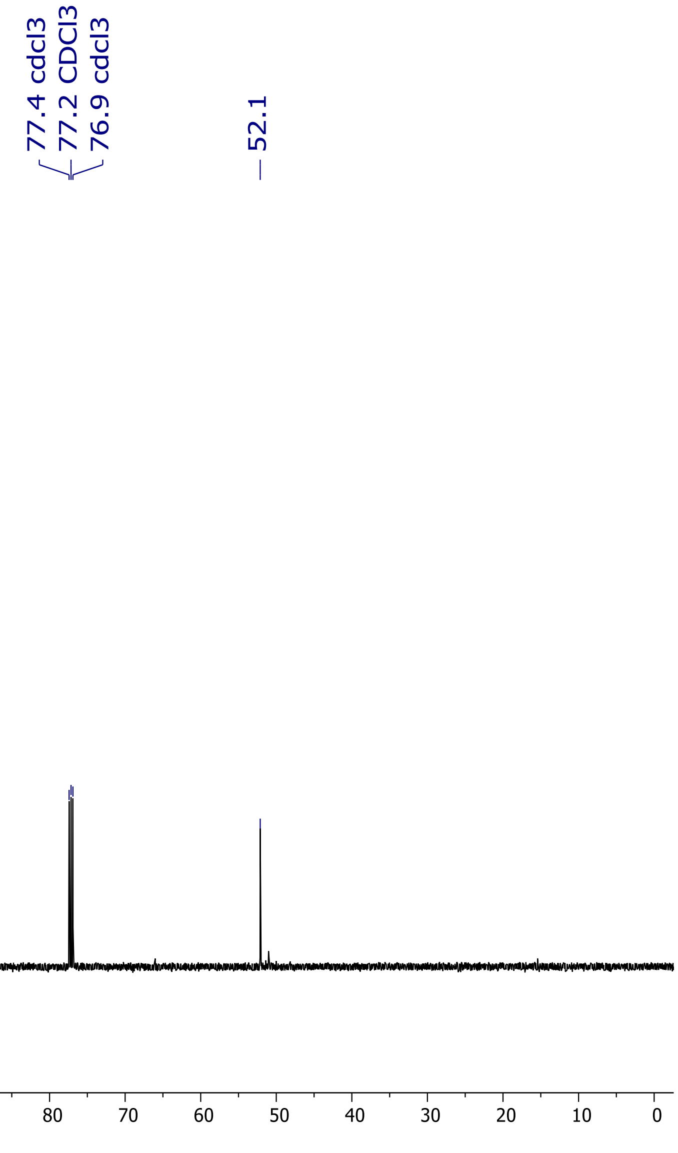

^{11}B NMR of *Tetramethoxydiboron* (2) ($CDCl_3$, 160 MHz)

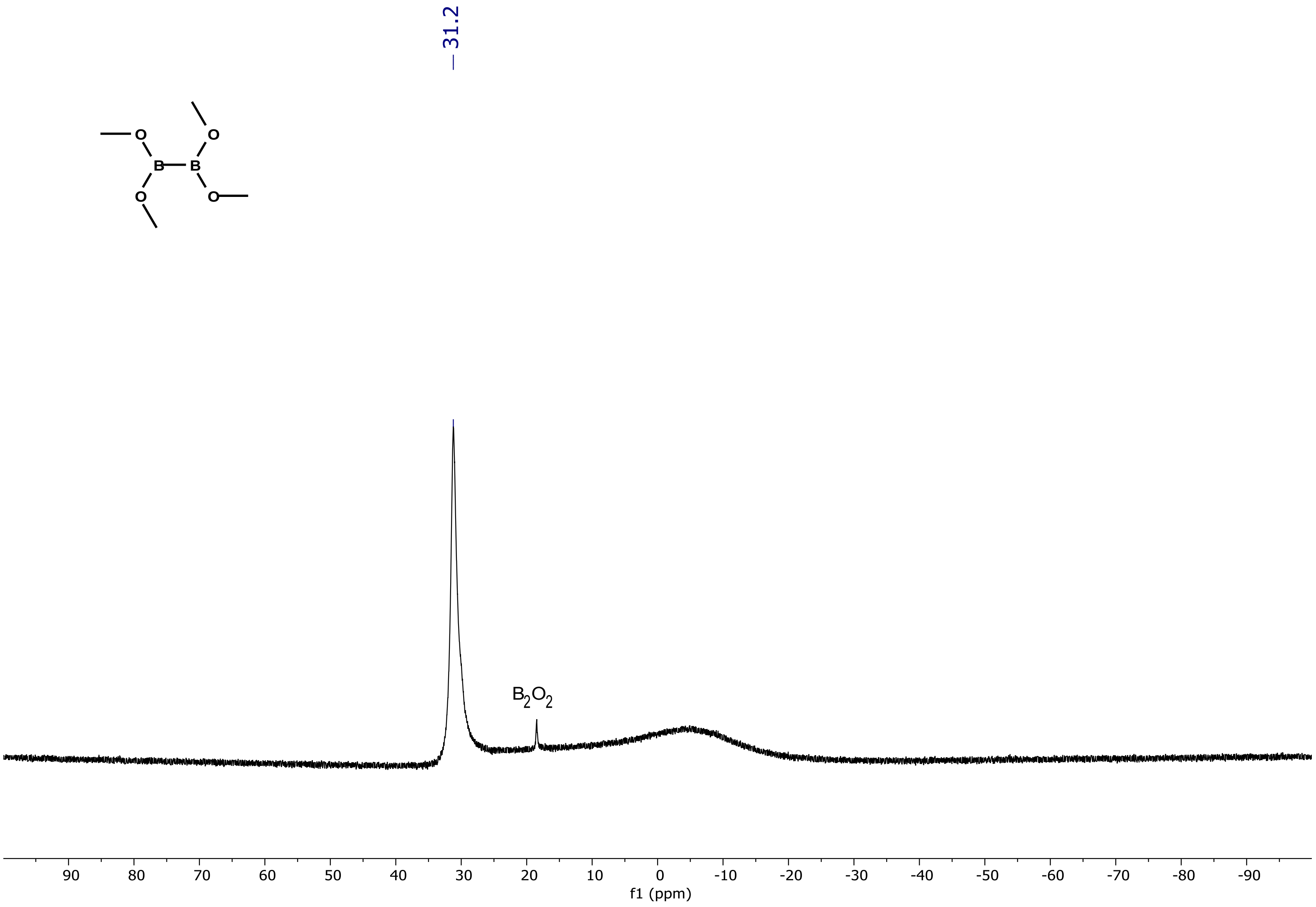

^{1}H NMR of *2,2'-Bi(1,3,2-dioxaborolane)* (B_2eg_2) (3) ($CDCl_3$, 500 MHz)

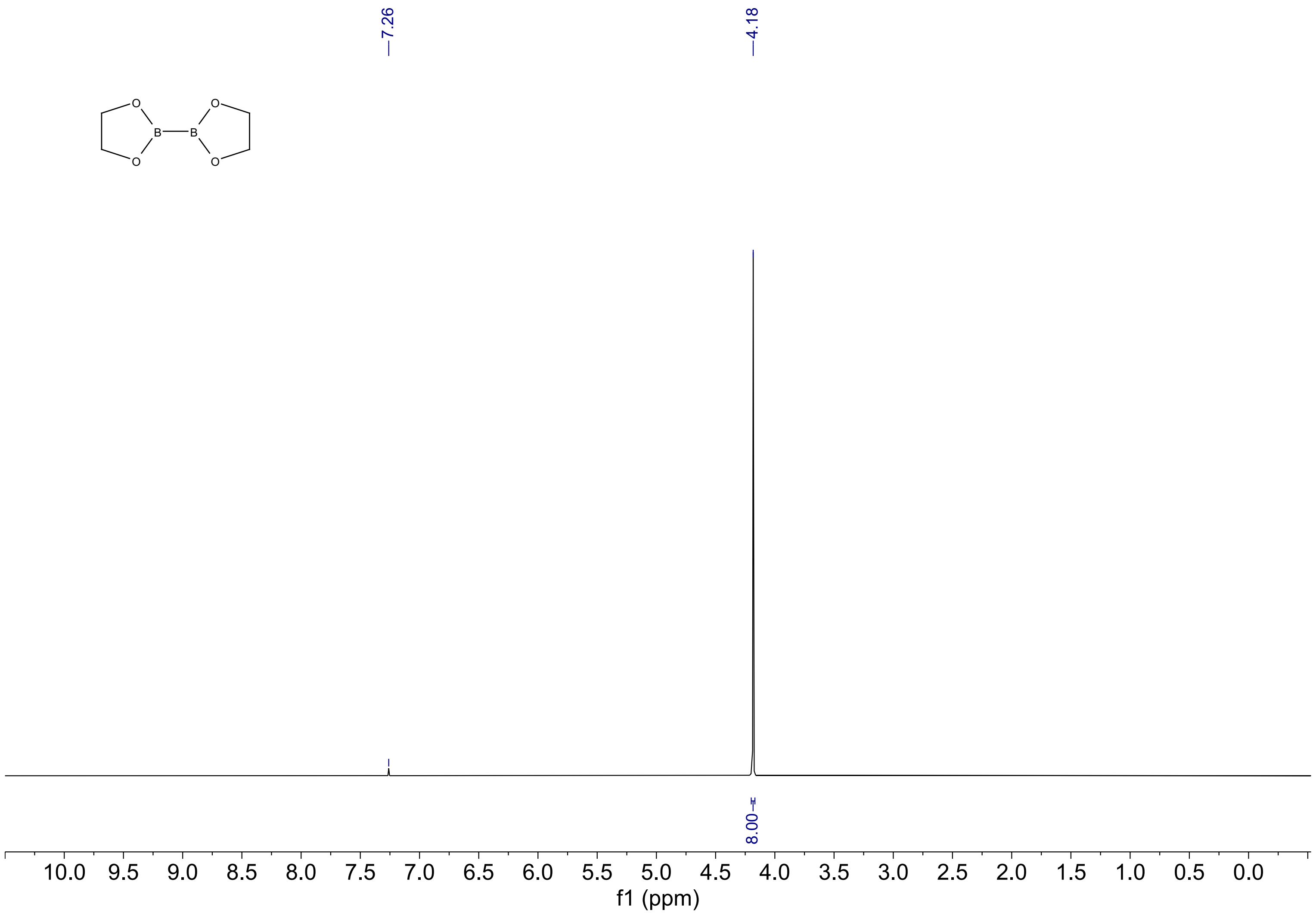

$^{13}C\{^{1}H\}$ NMR of *2,2'-Bi(1,3,2-dioxaborolane)* (B_2eg_2) (3) ($CDCl_3$, 126 MHz)

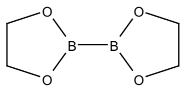

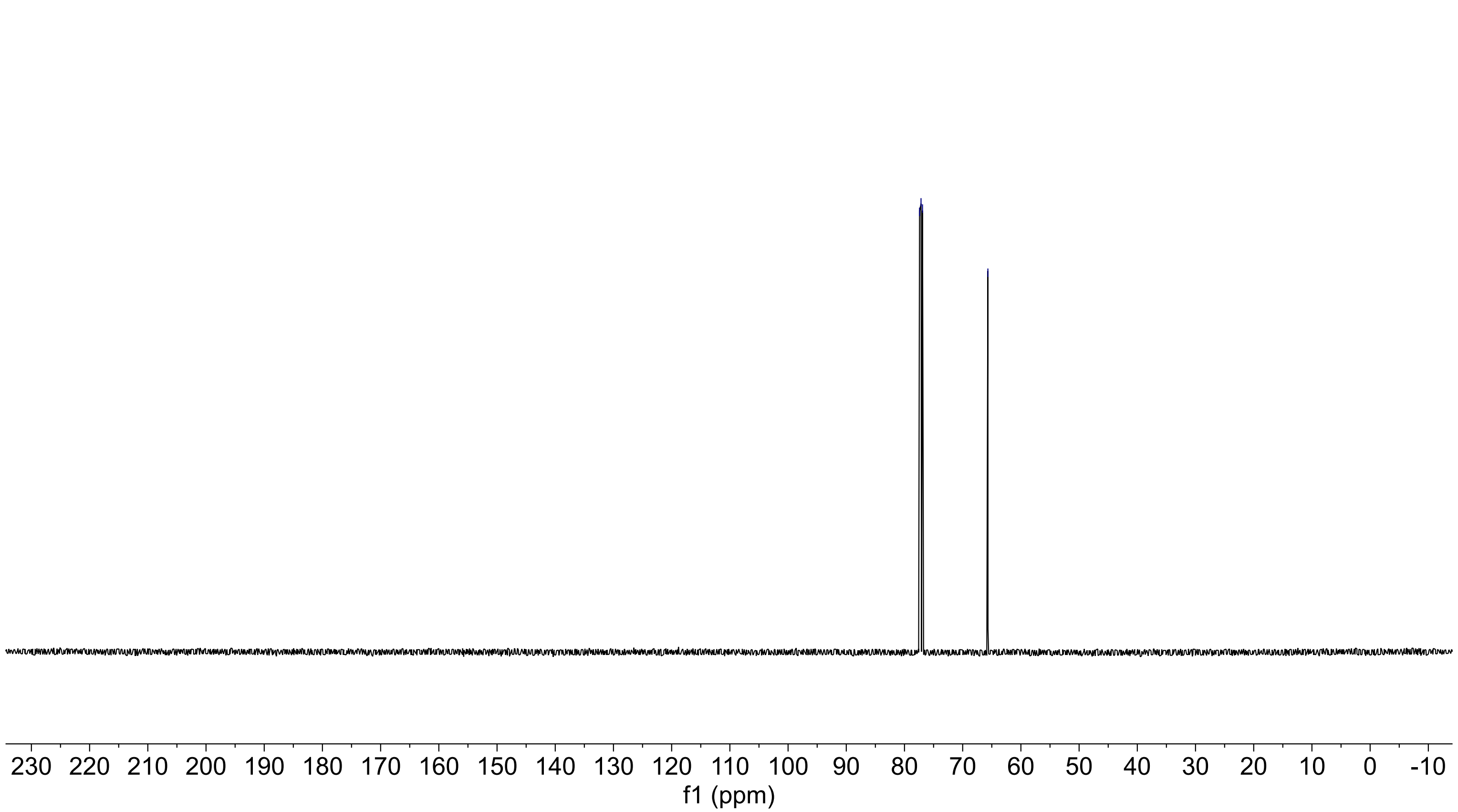

^{11}B NMR of *2,2'-Bi(1,3,2-dioxaborolane)* (B_2eg_2) (3) ($CDCl_3$, 160 MHz)

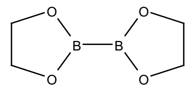

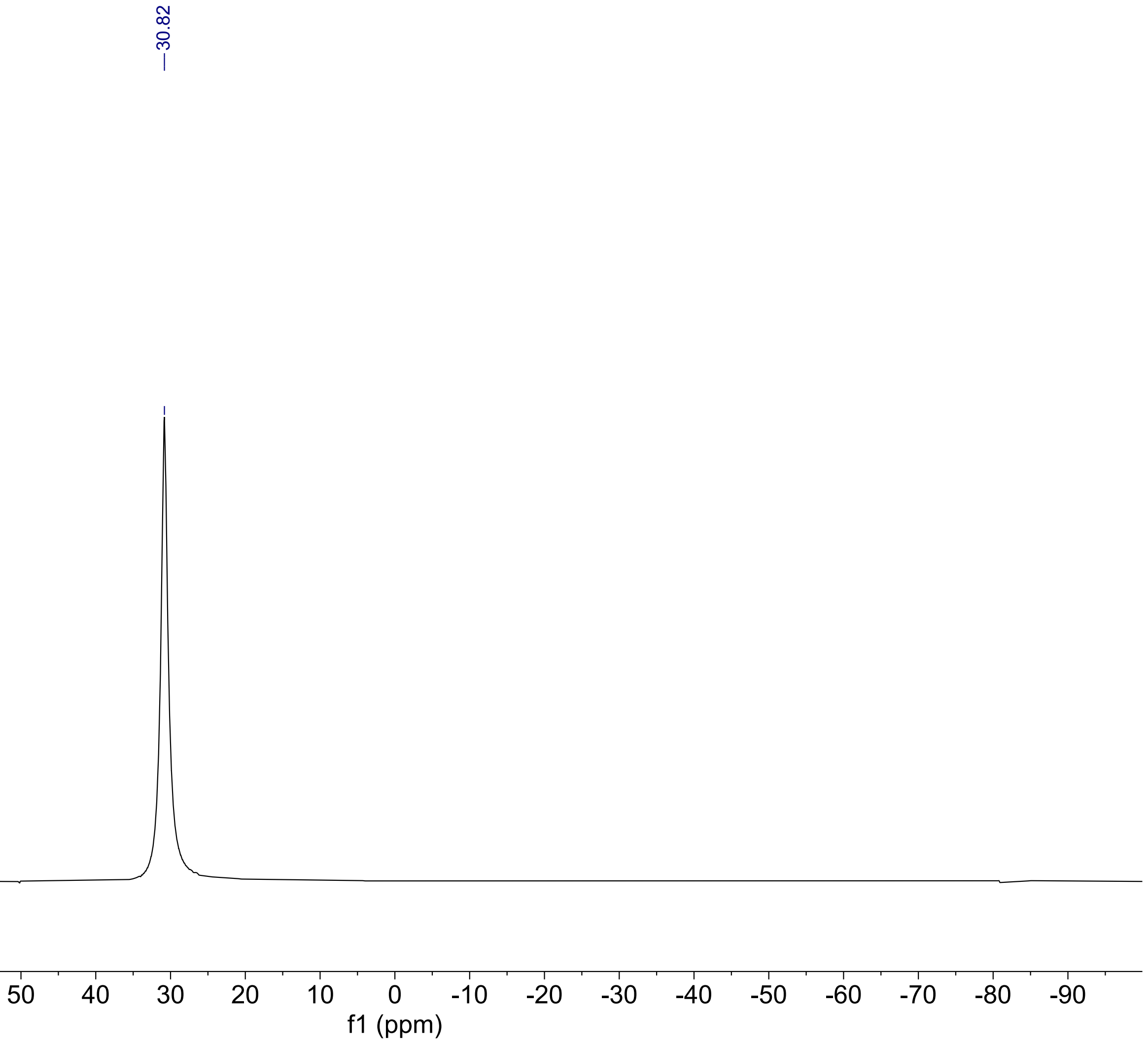

^{1}H NMR of *4,4'-Dimethyl-2,2'-bi(1,3,2-dioxaborolane) (B_2pg_2)* (4) ($CDCl_3$, 500 MHz)

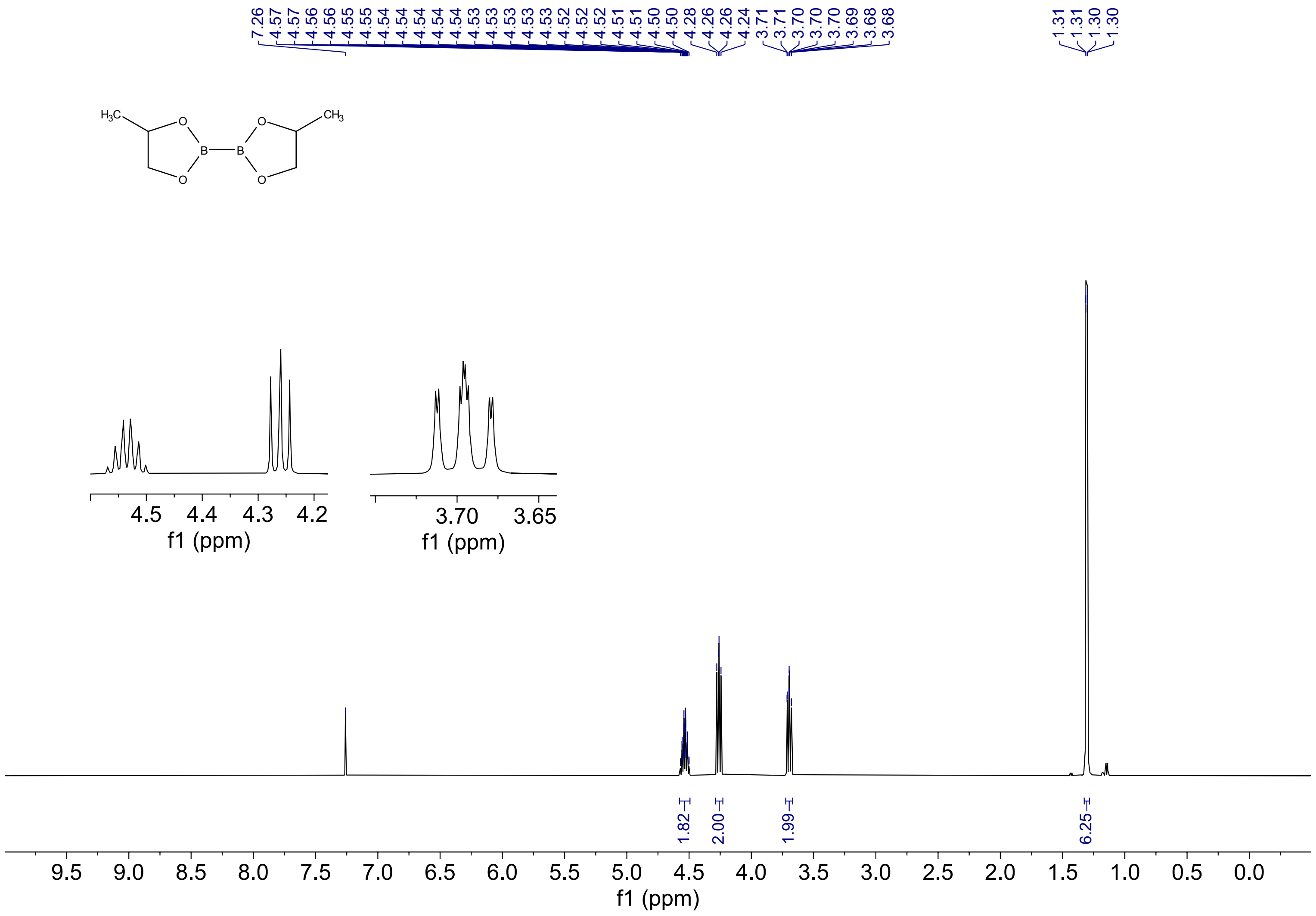

$^{13}C\{^{1}H\}$ NMR of *4,4'-Dimethyl-2,2'-bi(1,3,2-dioxaborolane) (B_2pg_2)* (4) ($CDCl_3$, 126 MHz)

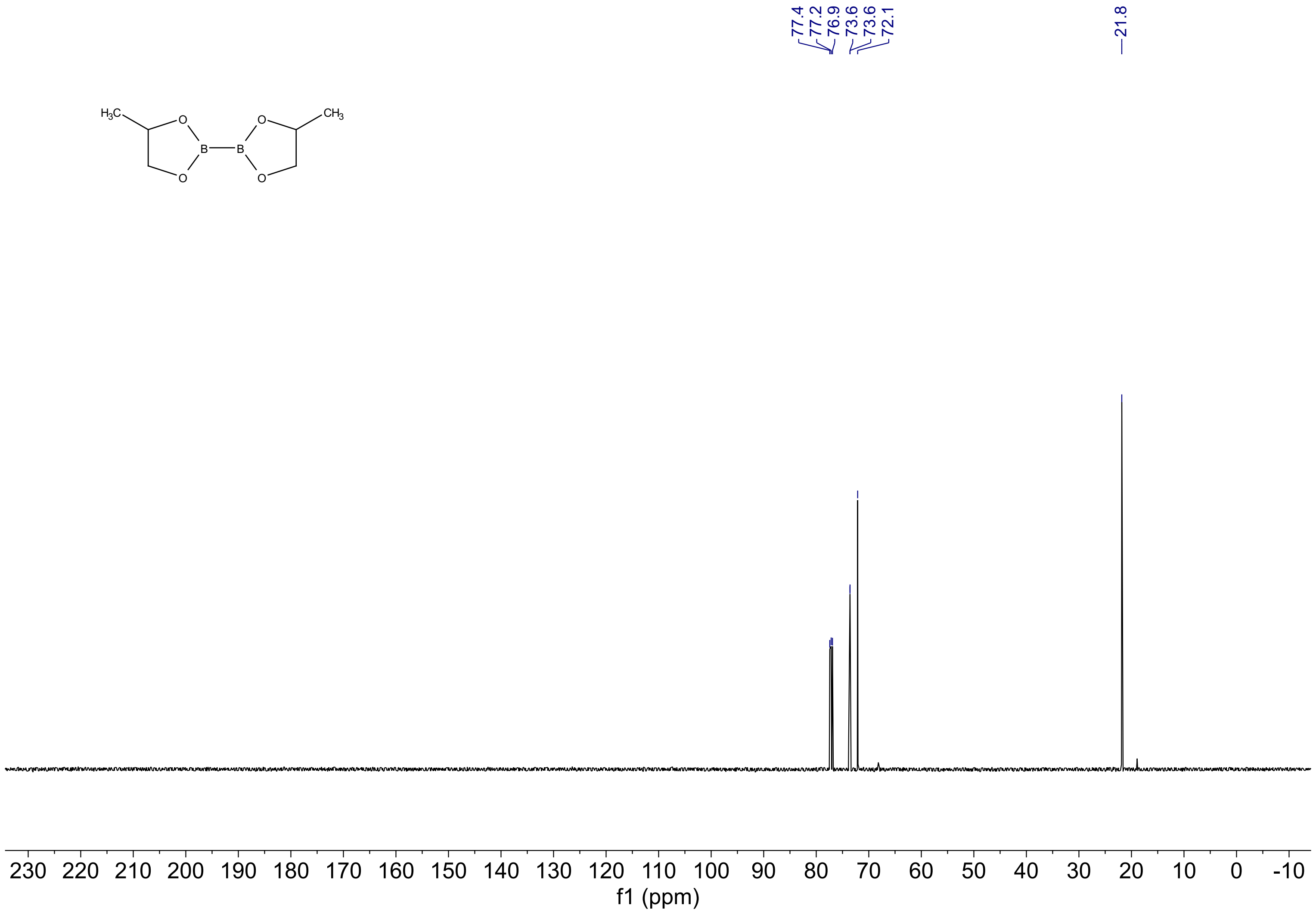

^{11}B NMR of *4,4'-Dimethyl-2,2'-bi(1,3,2-dioxaborolane) (B_2pg_2)* (4) ($CDCl_3$, 160 MHz)

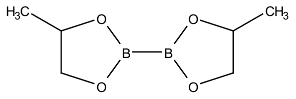

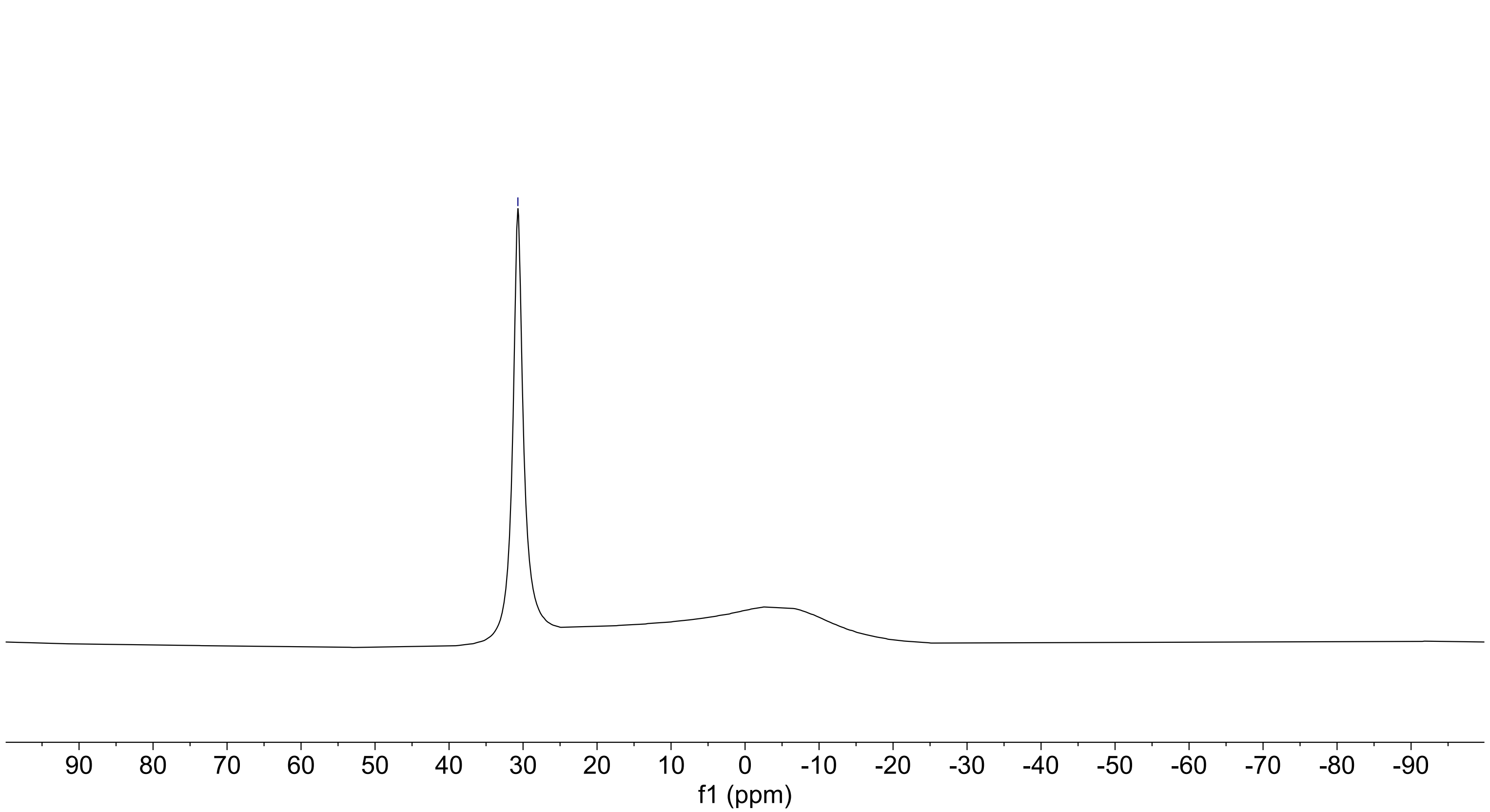

^{1}H NMR of *(4S,4'S) 4,4'-Dimethyl-2,2'-bi(1,3,2-dioxaborolane)*- ((S,S)-B_2pg_2) (5) ($CDCl_3$, 500 MHz)

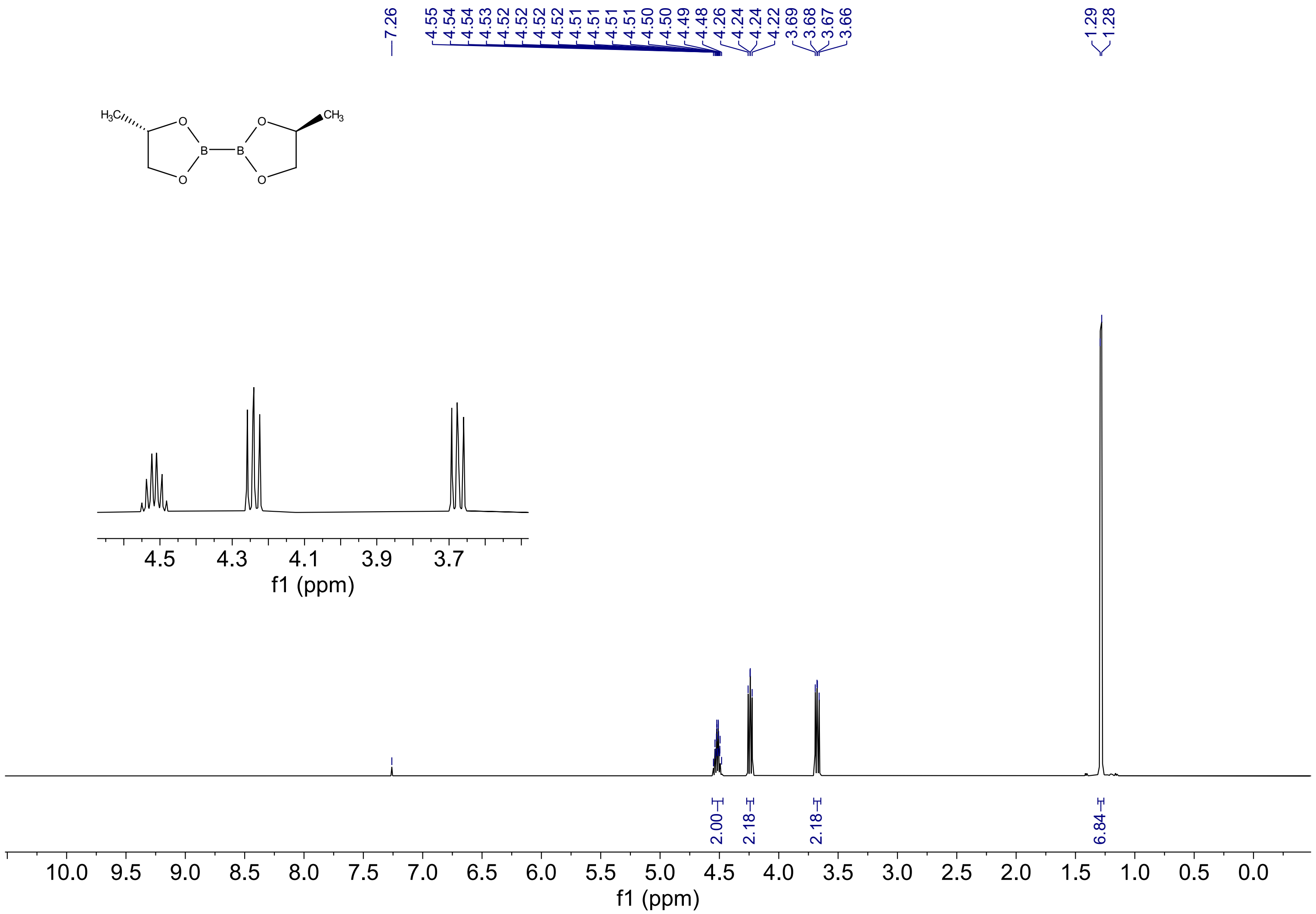

$^{13}C\{^1H\}$ NMR of *(4S,4'S) 4,4'-Dimethyl-2,2'-bi(1,3,2-*dioxaborolane)- ((S,S)-B_2pg_2) (5) ($CDCl_3$, 126 MHz)

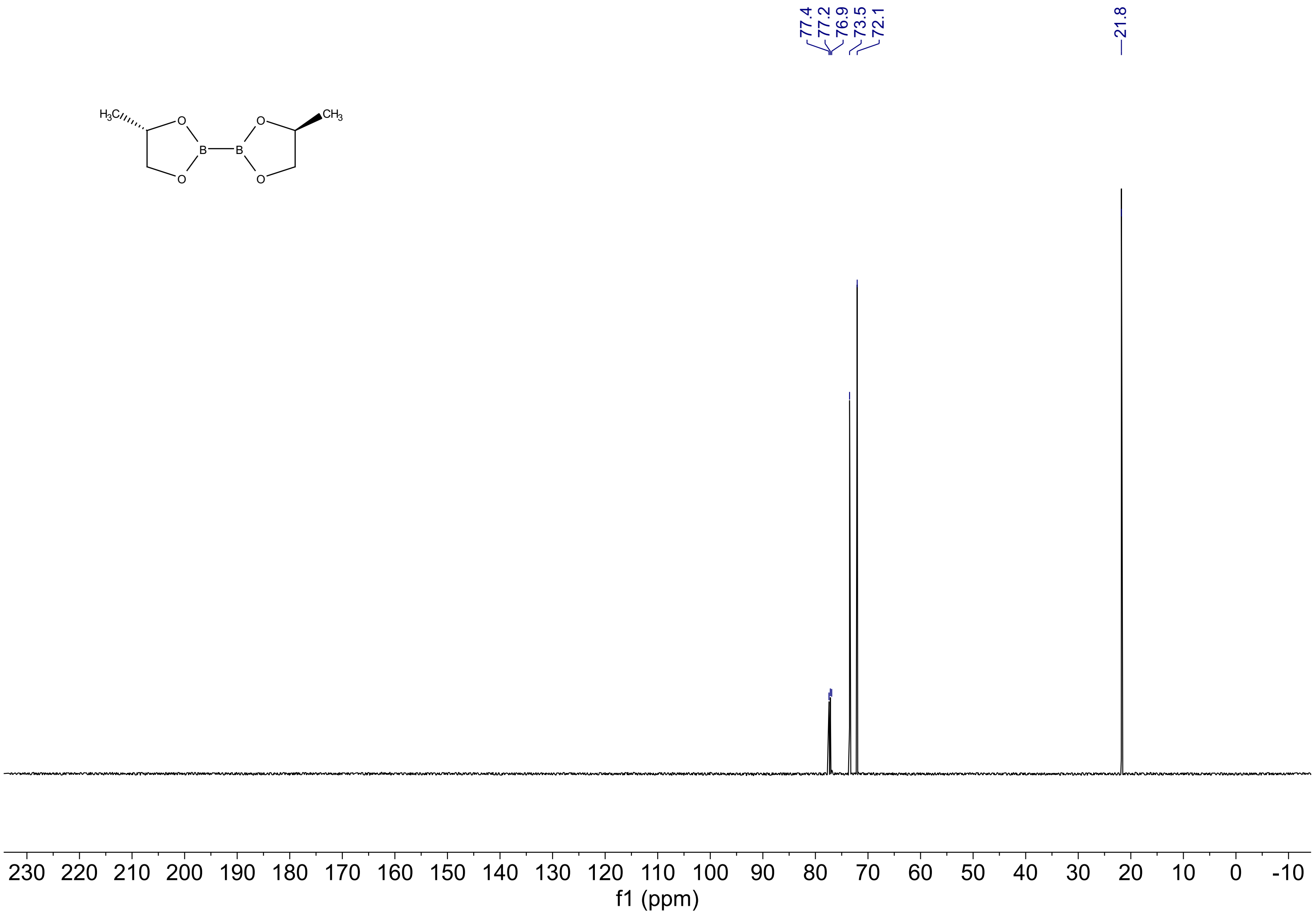

^{11}B NMR of *(4S,4'S) 4,4'-Dimethyl-2,2'-bi(1,3,2-dioxaborolane)* ((S,S)-B_2pg_2) (5) ($CDCl_3$, 160 MHz)

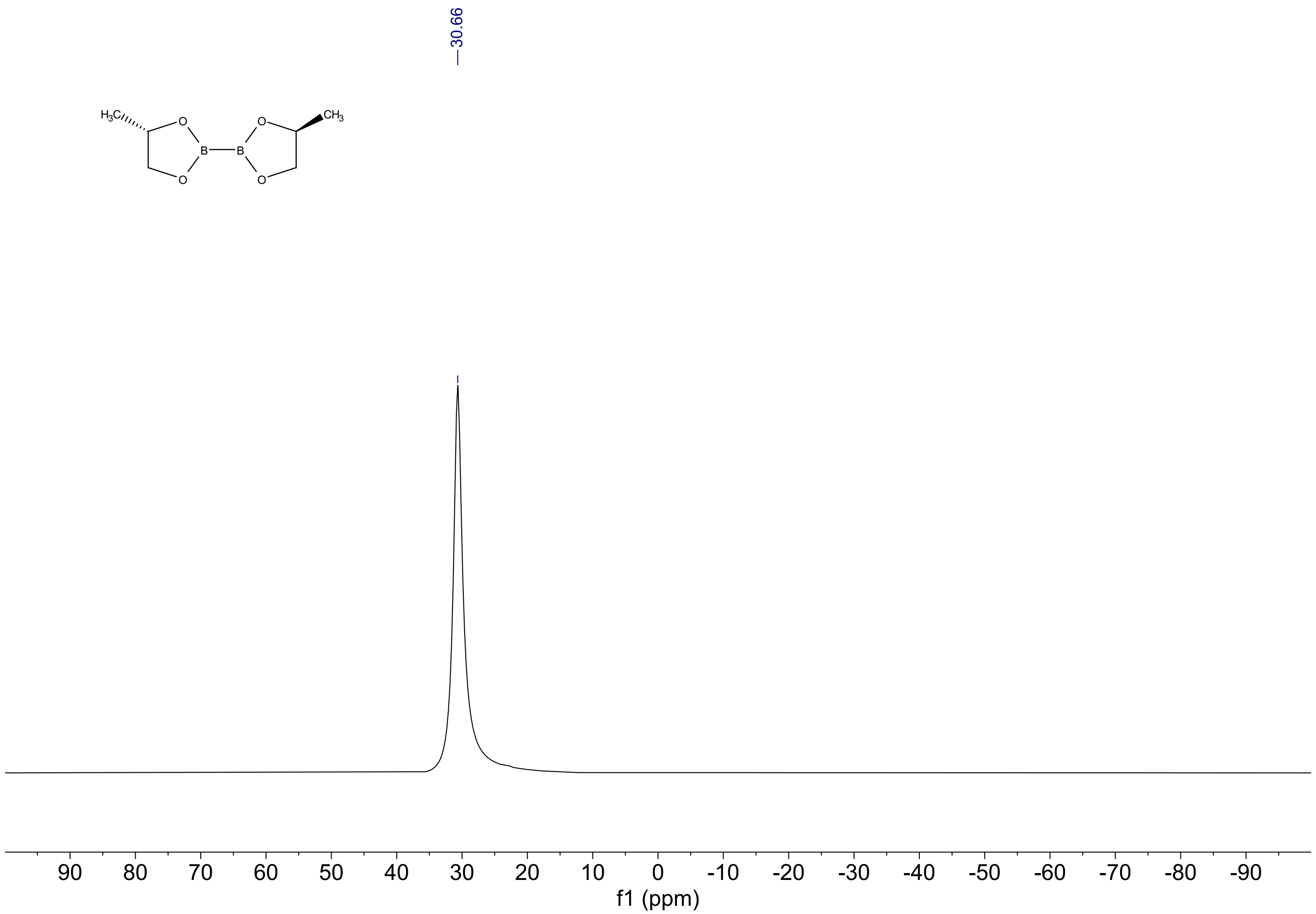

^{1}H NMR of *4,4'-Diethyl-2,2'-bi(1,3,2-dioxaborolane)* (B_2bg_2) (6) ($CDCl_3$, 500 MHz)

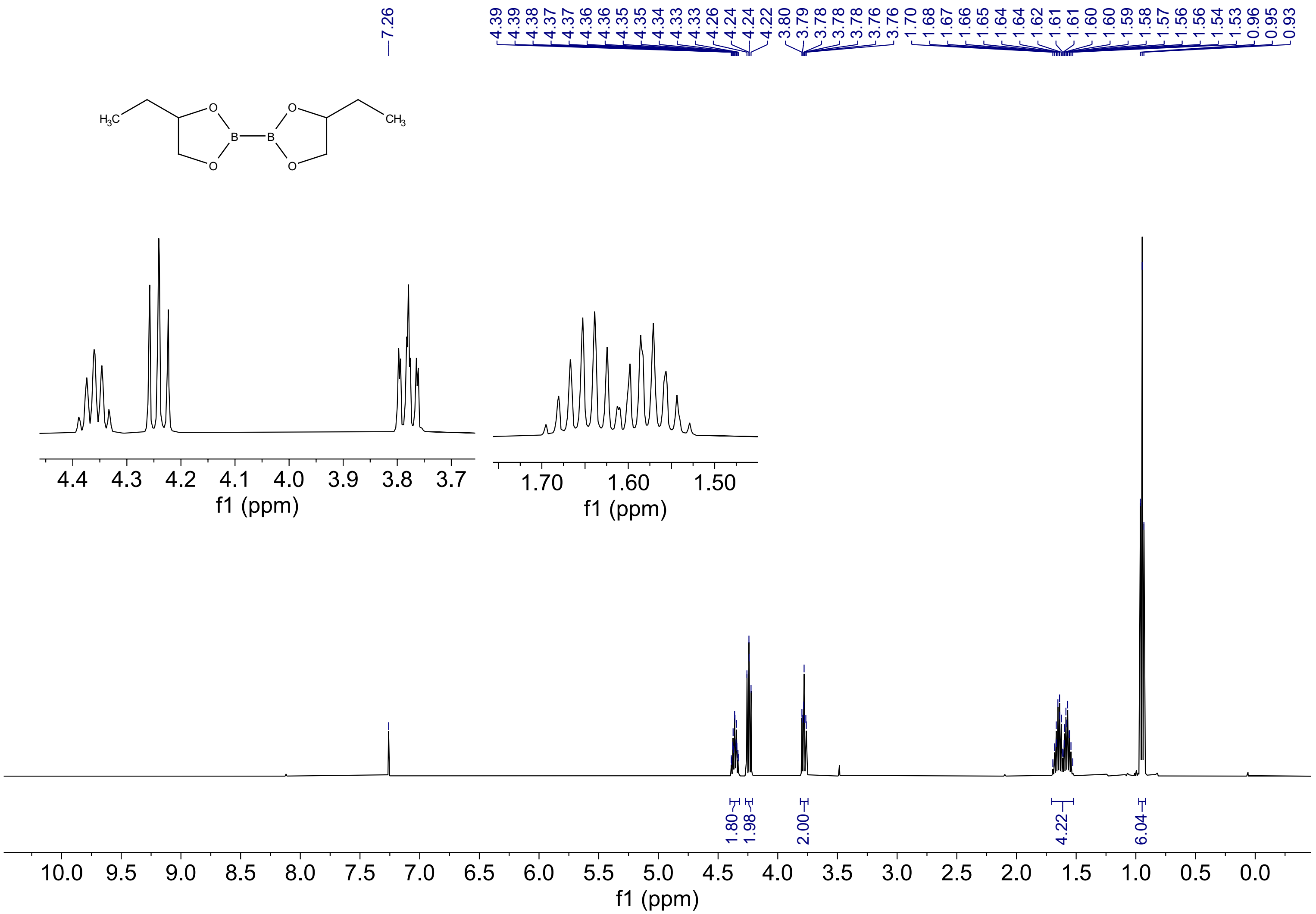

$^{13}C\{^{1}H\}$ NMR of *4,4'-Diethyl-2,2'-bi(1,3,2-dioxaborolane)* (B_2bg_2) (6) ($CDCl_3$, 126 MHz)

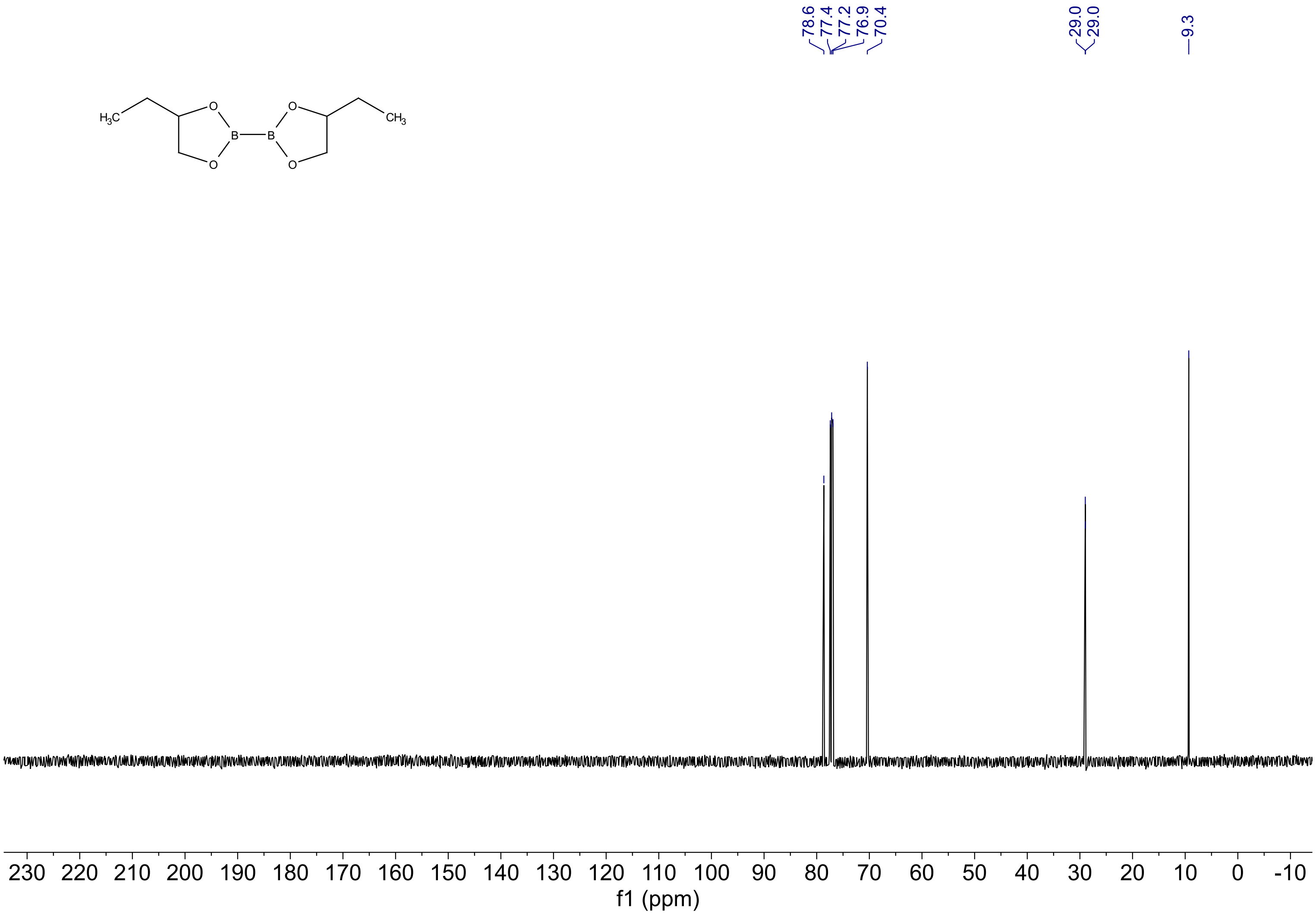

^{11}B NMR of *4,4'-Diethyl-2,2'-bi(1,3,2-dioxaborolane)* (B_2bg_2) (6) ($CDCl_3$, 160 MHz)

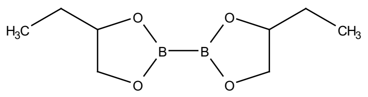

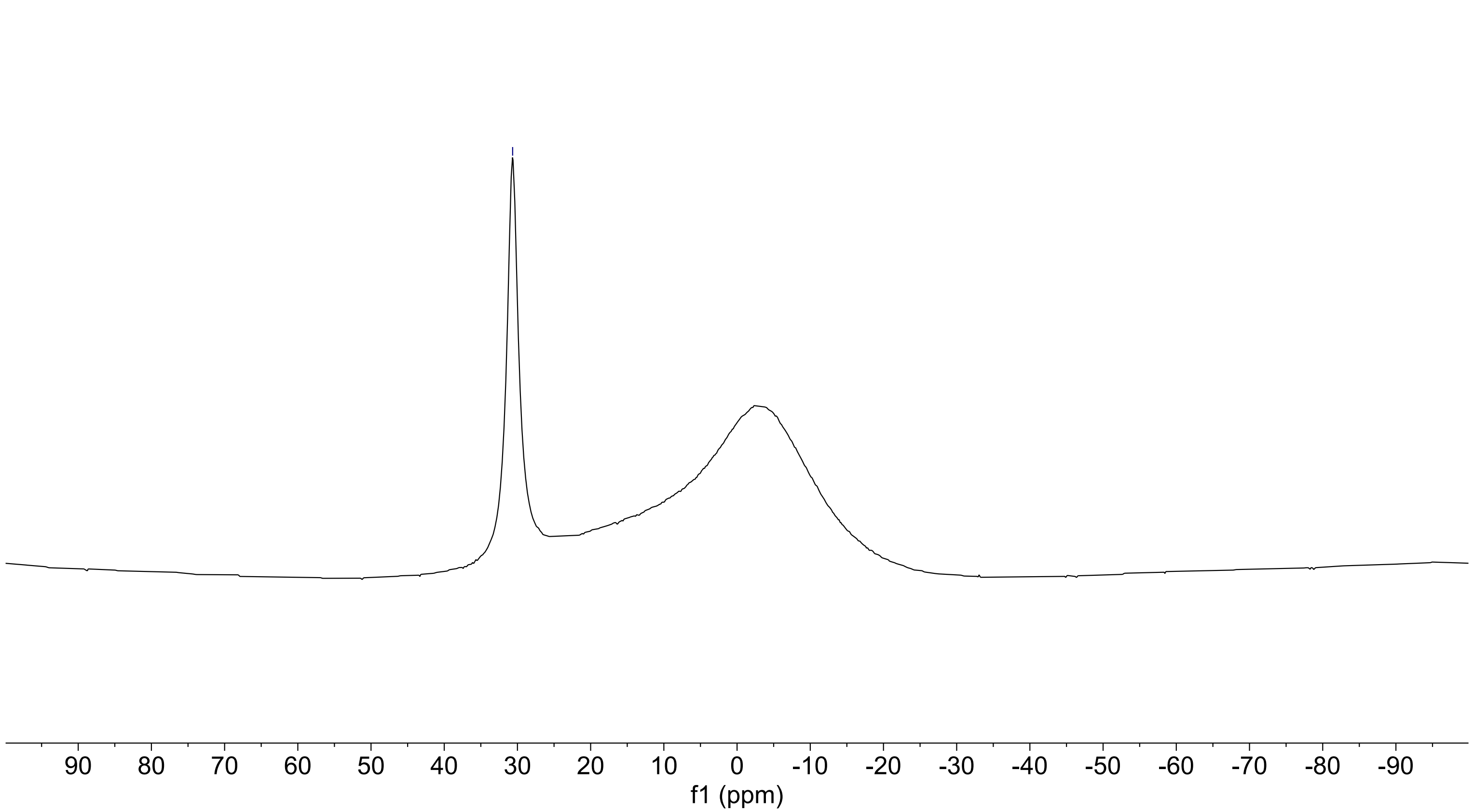

^{1}H NMR of *4,4,4',4'-Tetramethyl-2,2'-bi(1,3,2-dioxaborolane)* (7) ($CDCl_3$, 500 MHz)

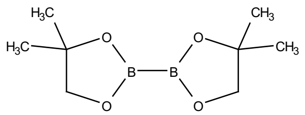

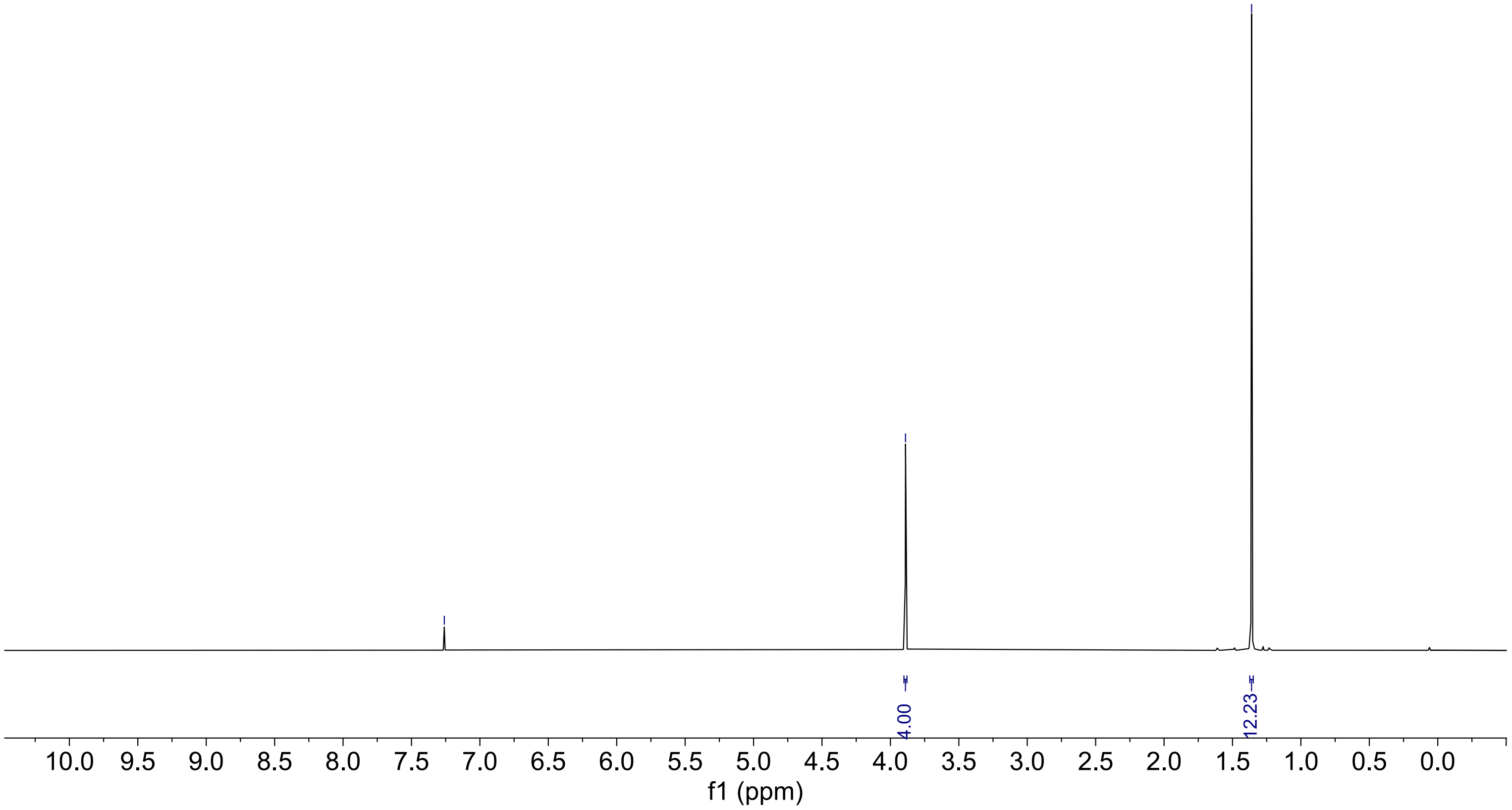

$^{13}C\{^{1}H\}$ NMR *4,4,4',4'-Tetramethyl-2,2'-bi(1,3,2-dioxaborolane)* (7) ($CDCl_3$, 126 MHz)

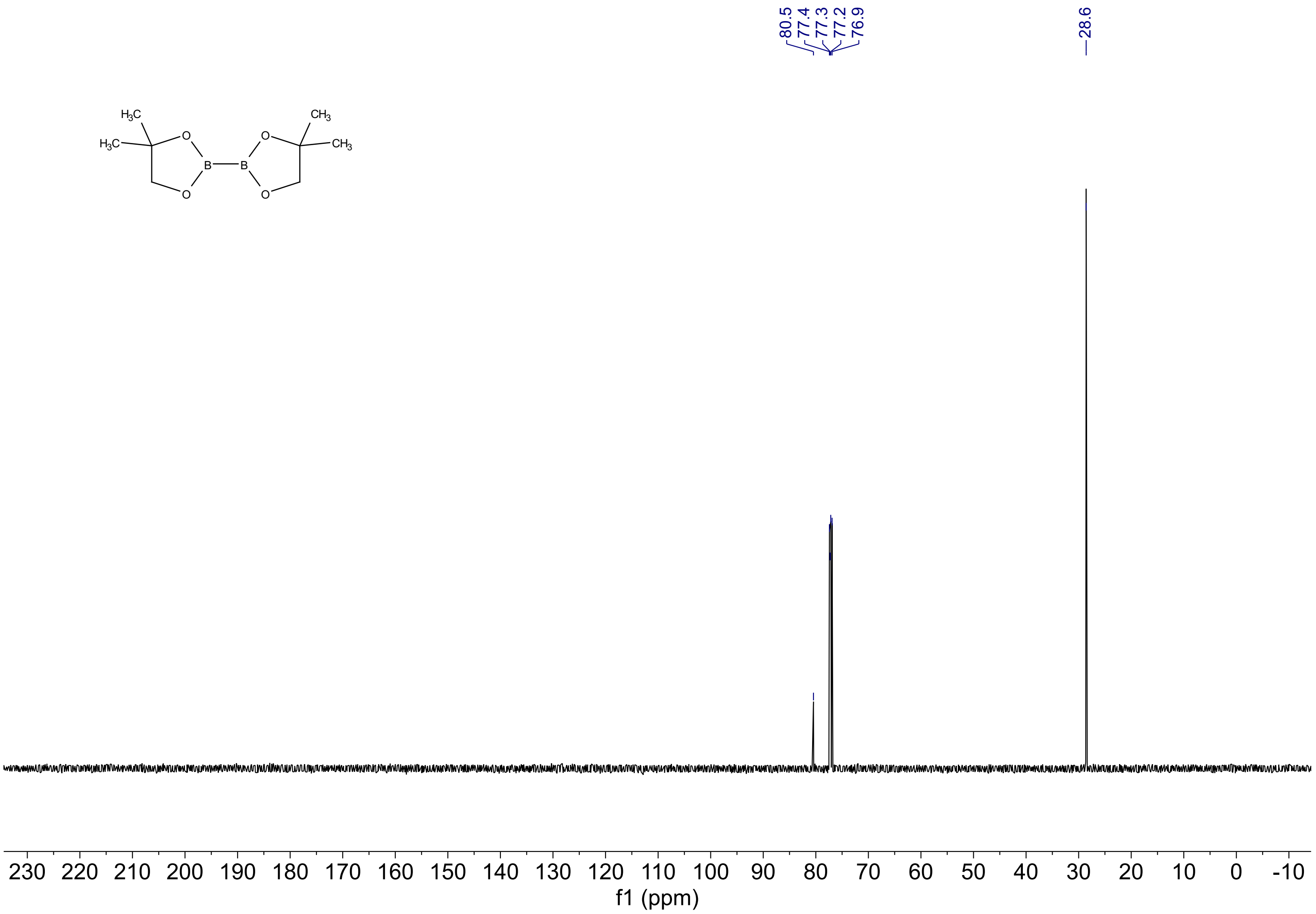

^{11}B NMR of *4,4,4',4'-Tetramethyl-2,2'-bi(1,3,2-dioxaborolane)* (7) ($CDCl_3$, 160 MHz)

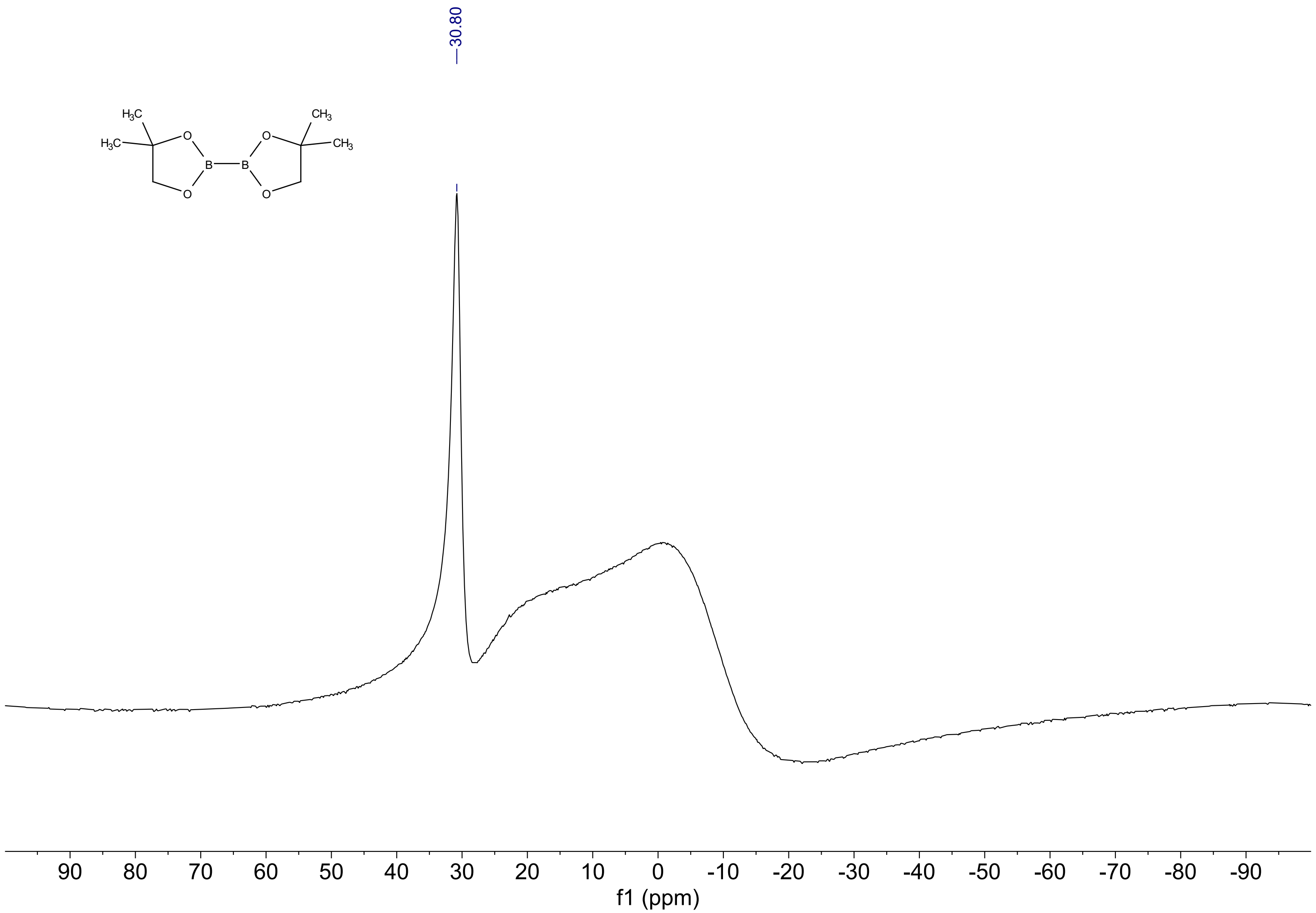

^{1}H NMR of *(4R,4'R,5R,5'R)-4,4',5,5'-Tetramethyl-2,2'-bi(1,3,2-dioxaborolane)* (8) ($CDCl_3$, 500 MHz)

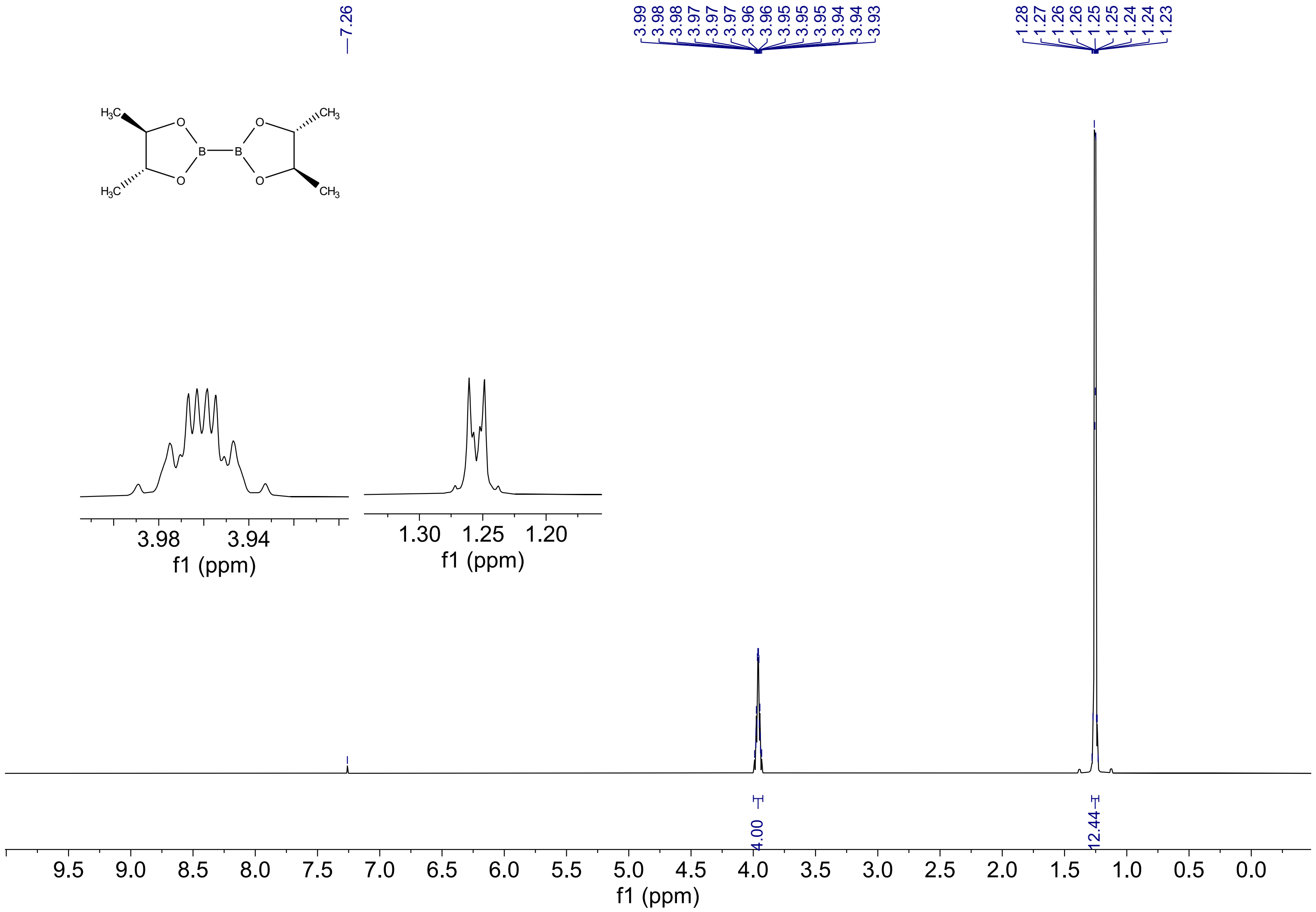

$^{13}C\{^{1}H\}$ NMR *(4R,4'R,5R,5'R)-4,4',5,5'-Tetramethyl-2,2'-bi(1,3,2-dioxaborolane)* (8) ($CDCl_3$, 126 MHz)

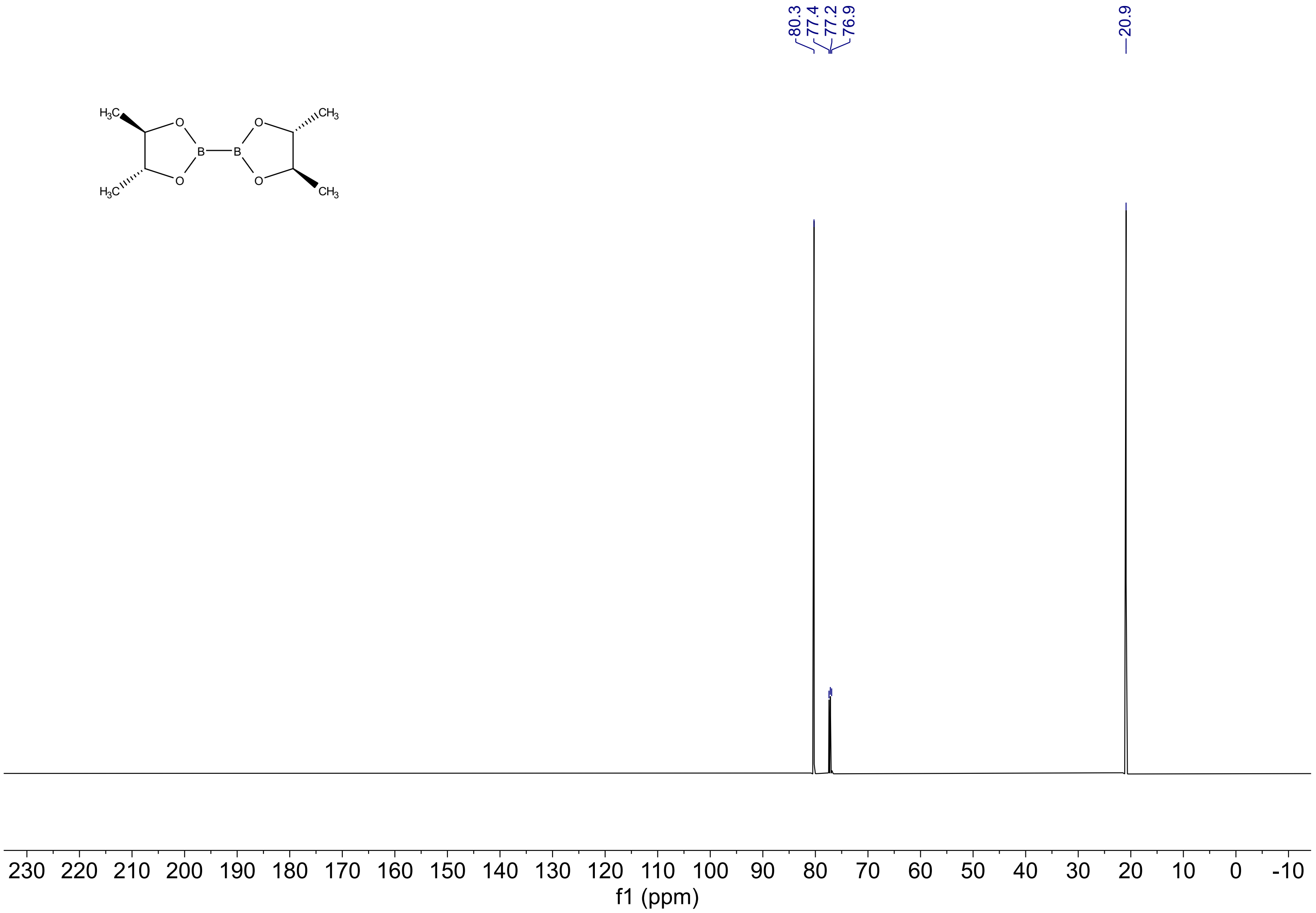

^{11}B NMR of *(4R,4'R,5R,5'R)-4,4',5,5'-Tetramethyl-2,2'-bi(1,3,2-dioxaborolane)* (8) ($CDCl_3$, 160 MHz)

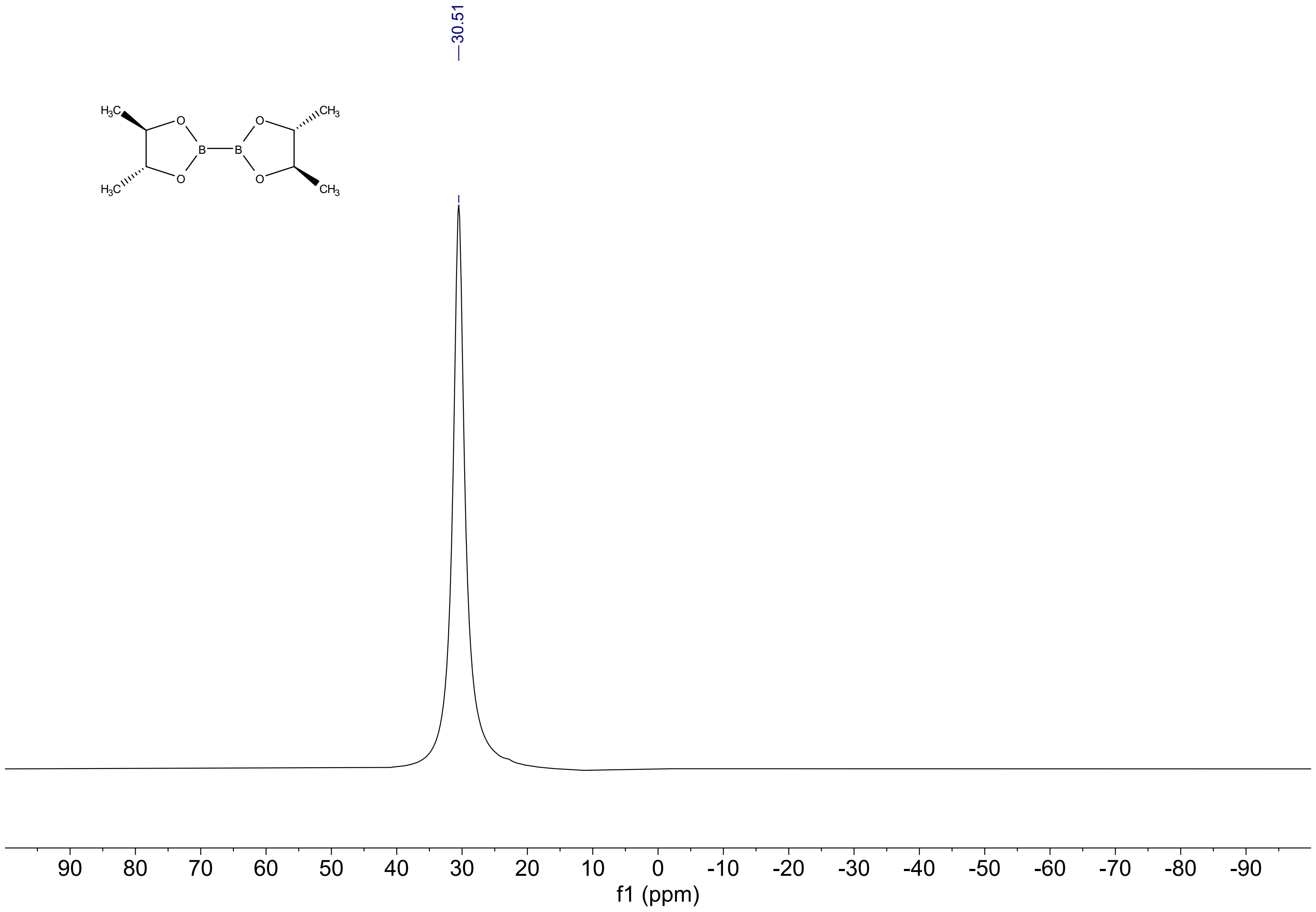

^{1}H NMR of *(4S,4'S,5S,5'S)-4,4',5,5'-Tetraphenyl-2,2'-bi(1,3,2-dioxaborolane)* (9) ($CDCl_3$, 500 MHz)

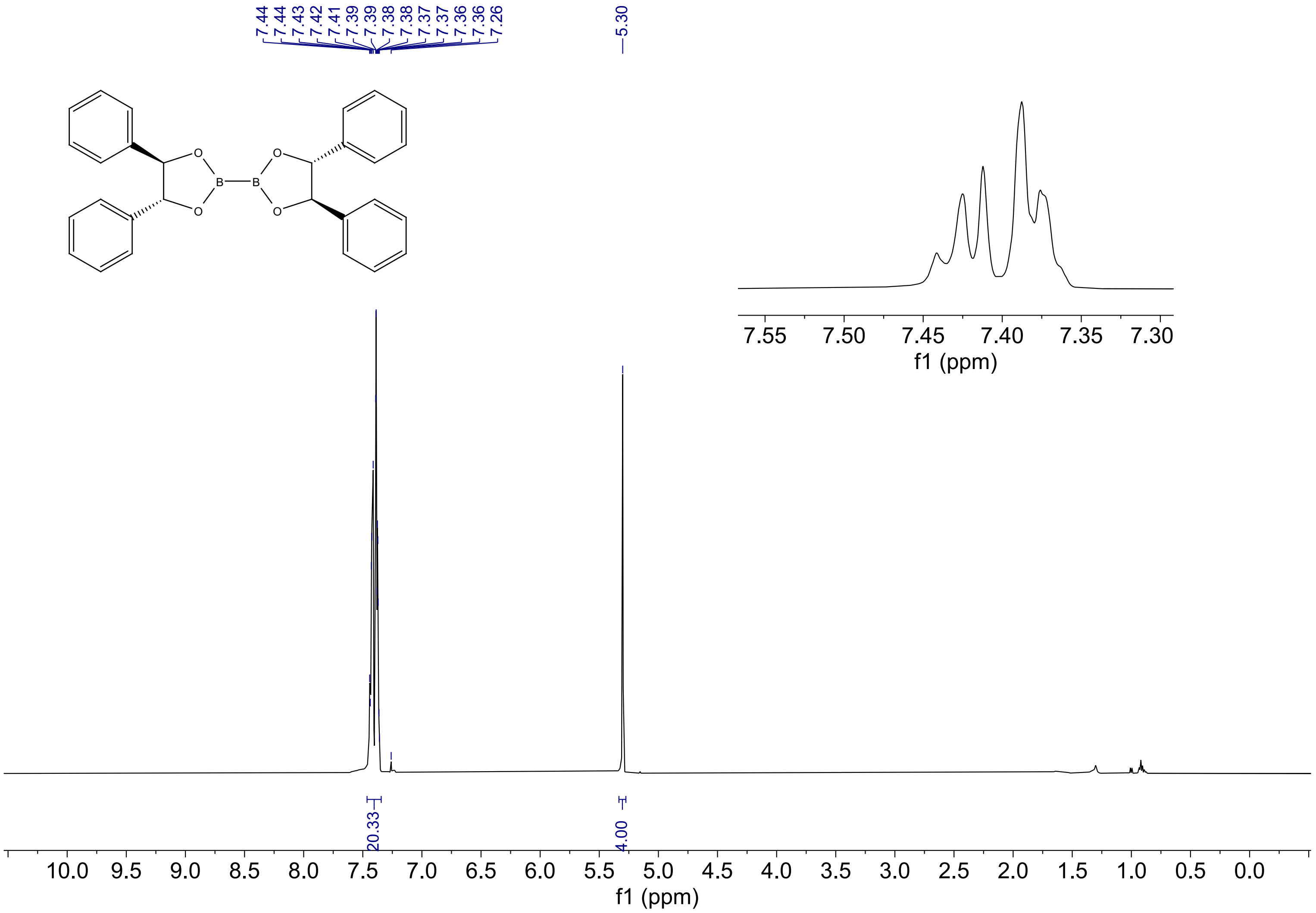

$^{13}C\{^{1}H\}$ NMR *(4S,4'S,5S,5'S)-4,4',5,5'-Tetraphenyl-2,2'-bi(1,3,2-dioxaborolane)* (9) ($CDCl_3$, 126 MHz)

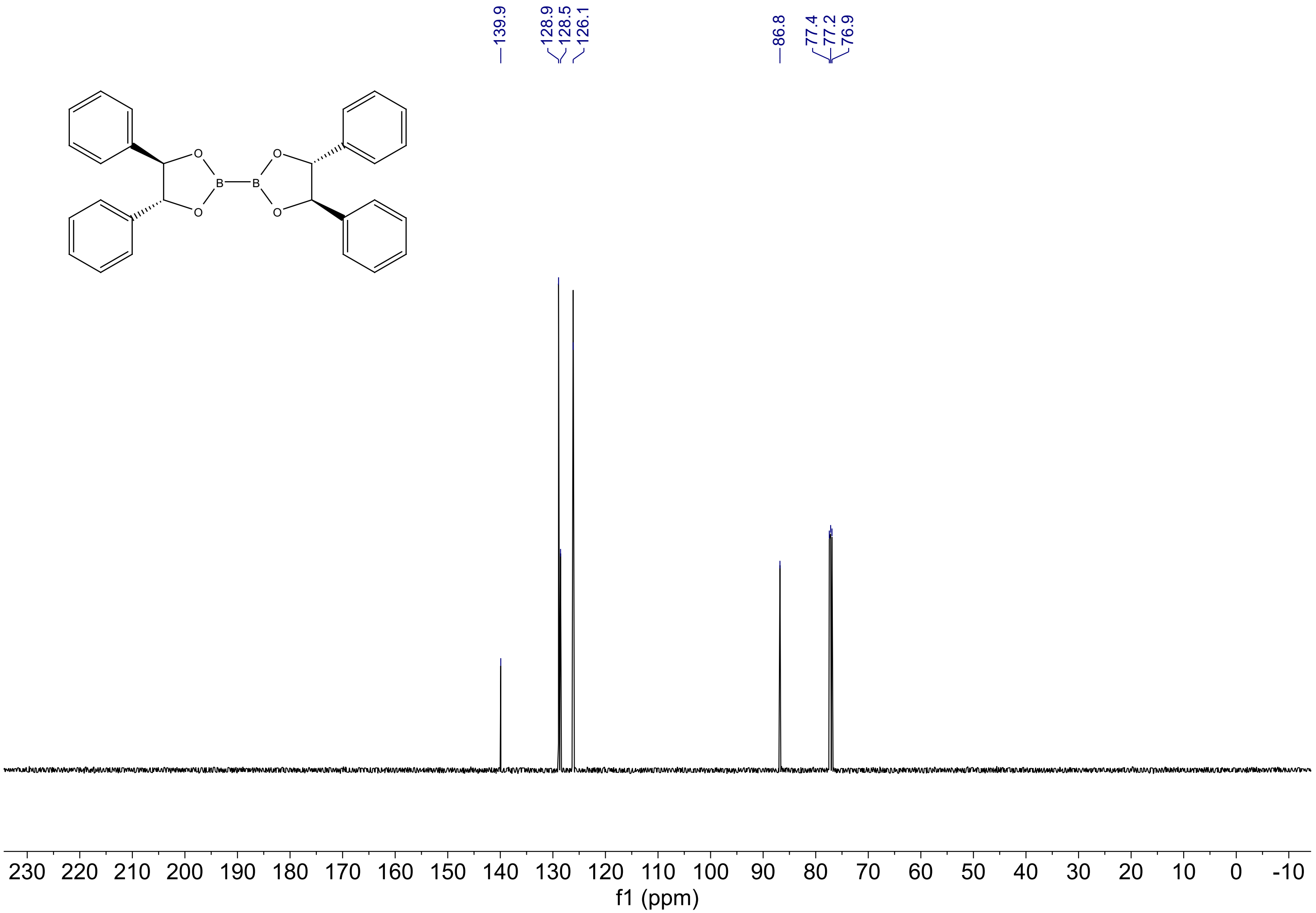

^{11}B NMR of *(4S,4'S,5S,5'S)-4,4',5,5'-Tetraphenyl-2,2'-bi(1,3,2-dioxaborolane)* (9) ($CDCl_3$, 160 MHz)

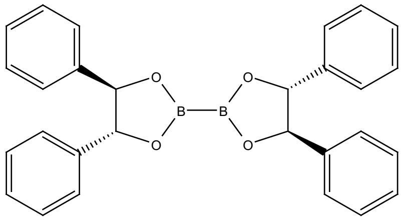

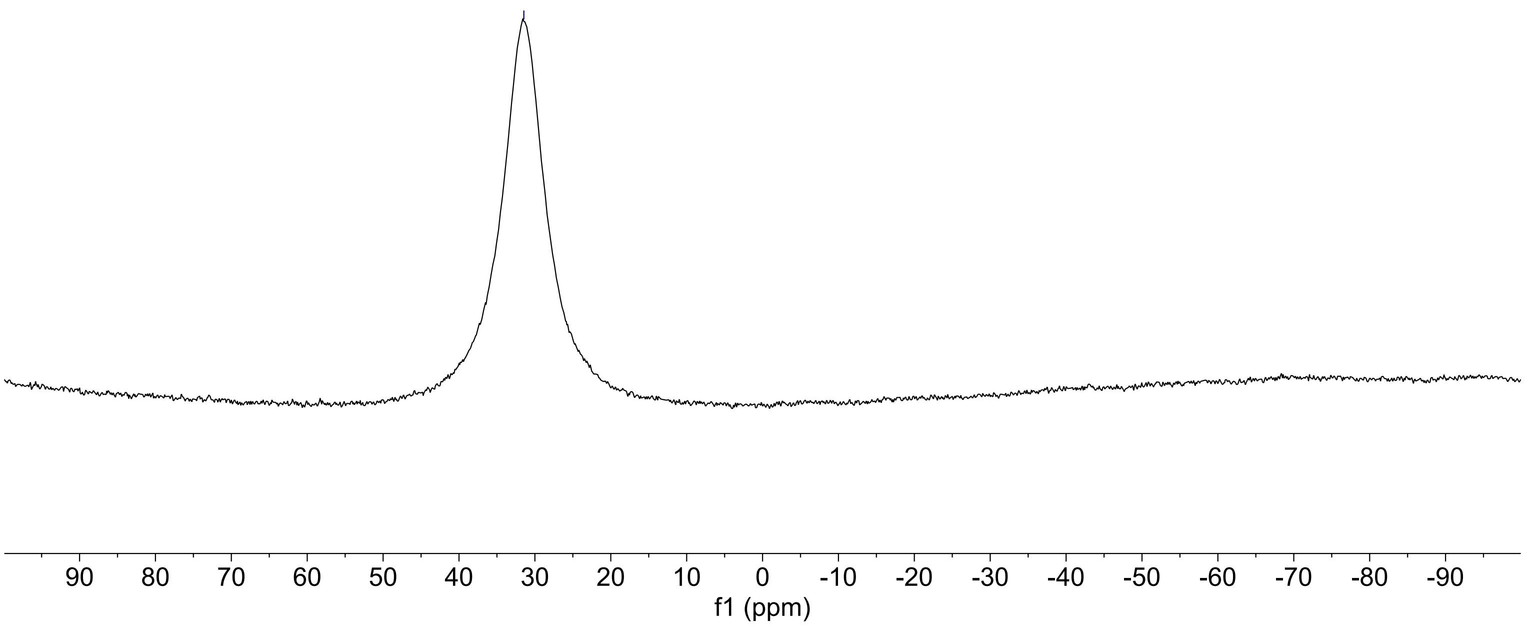

^{1}H NMR of *4,4'-Dimethyl-2,2'-bi(1,3,2-dioxaborinane)* (10) ($CDCl_3$, 500 MHz)

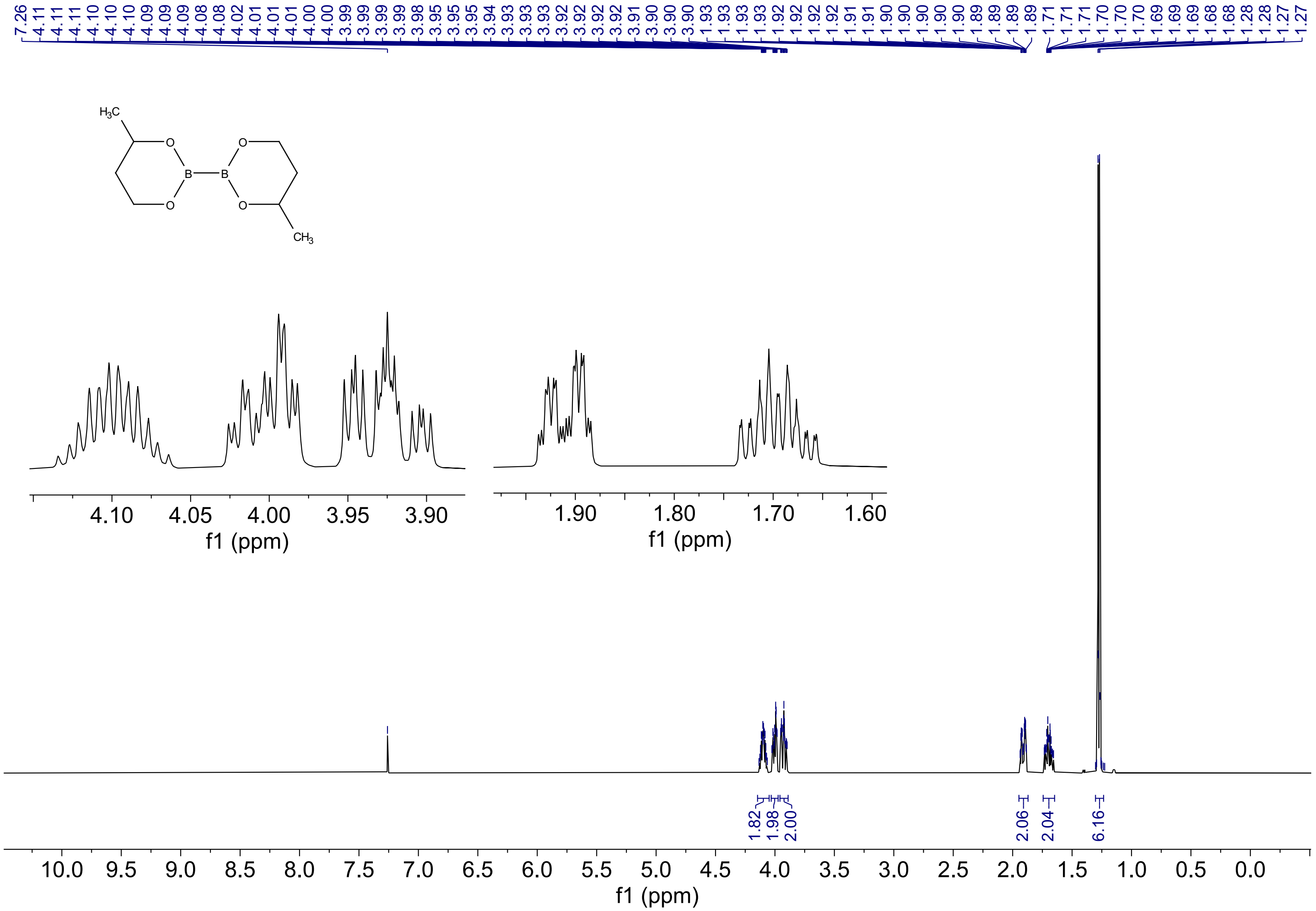

$^{13}C\{^{1}H\}$ NMR of *4,4'-Dimethyl-2,2'-bi(1,3,2-dioxaborinane)* (10) ($CDCl_3$, 126 MHz)

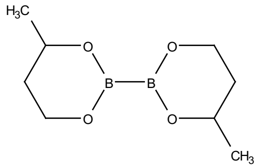

^{11}B NMR of *4,4'-Dimethyl-2,2'-bi(1,3,2-dioxaborinane)* (10) ($CDCl_3$, 160 MHz)

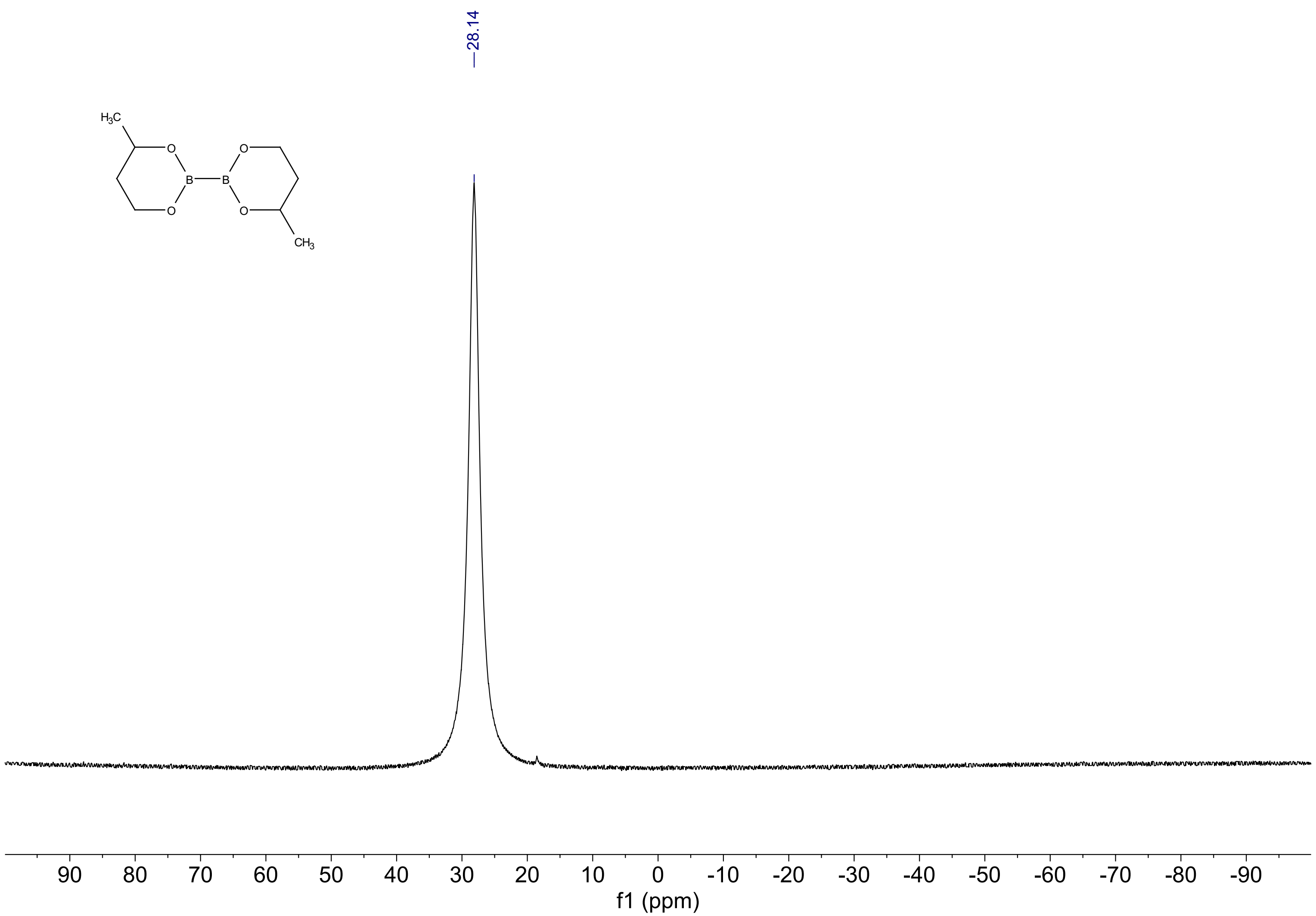

^{1}H NMR of *5,5,5′,5′-Tetramethyl-2,2′-bi(1,3,2-dioxaborinane)* (11) ($CDCl_3$, 500 MHz)

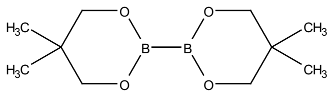

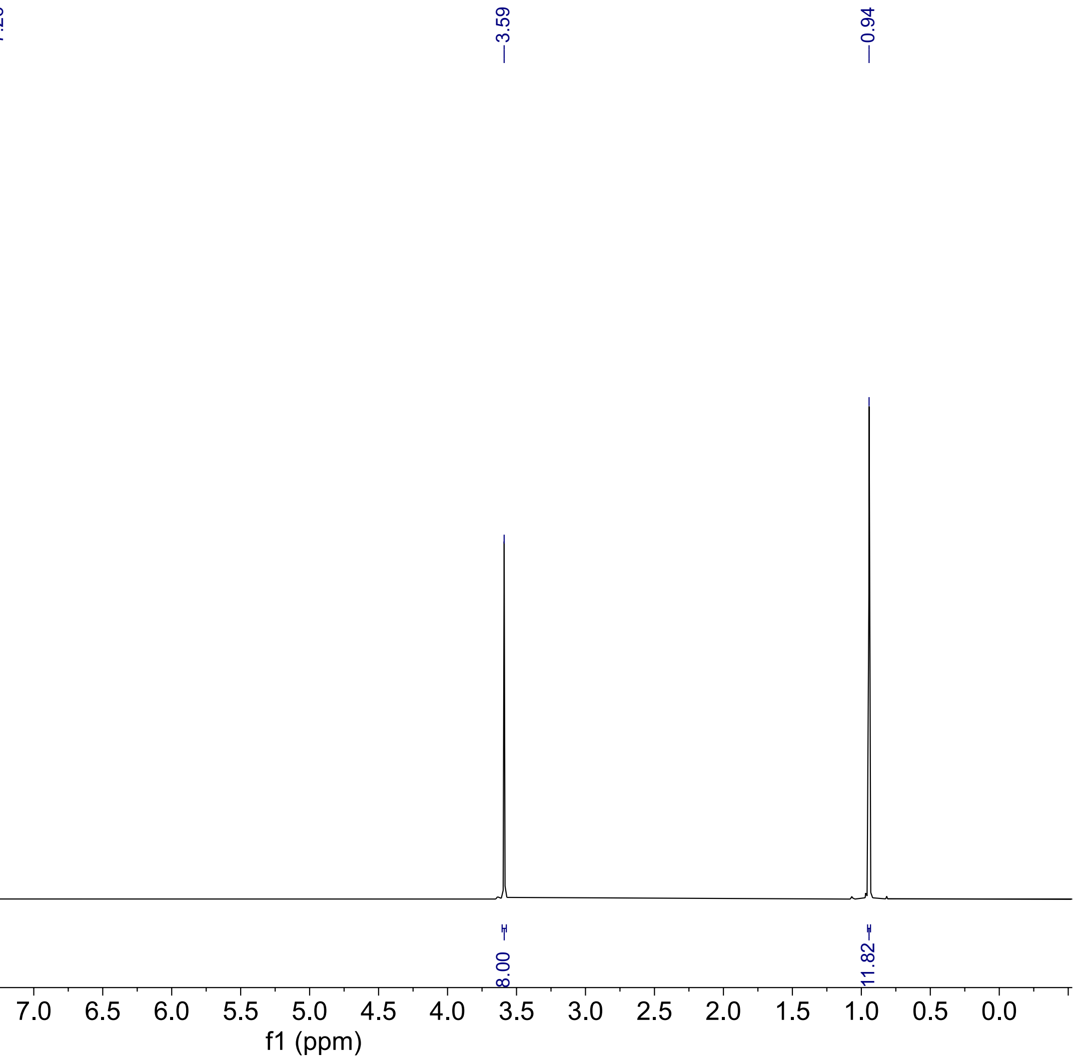

$^{13}C\{^{1}H\}$ NMR of *5,5,5',5'-Tetramethyl-2,2'-bi(1,3,2-dioxaborinane)* (11) ($CDCl_3$, 126 MHz)

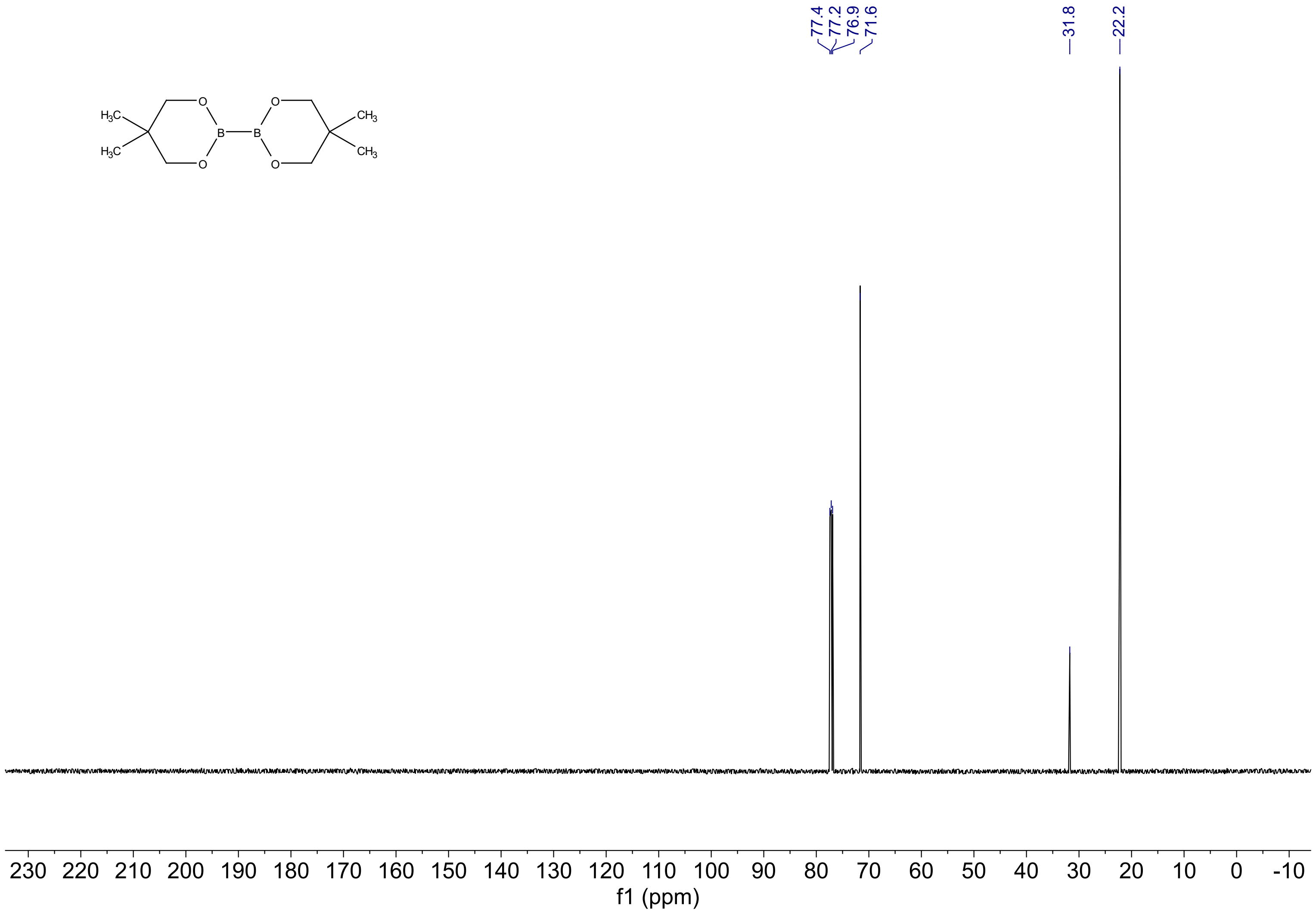

^{11}B NMR of *5,5,5′,5′-Tetramethyl-2,2′-bi(1,3,2-dioxaborinane)* (11) ($CDCl_3$, 160 MHz)

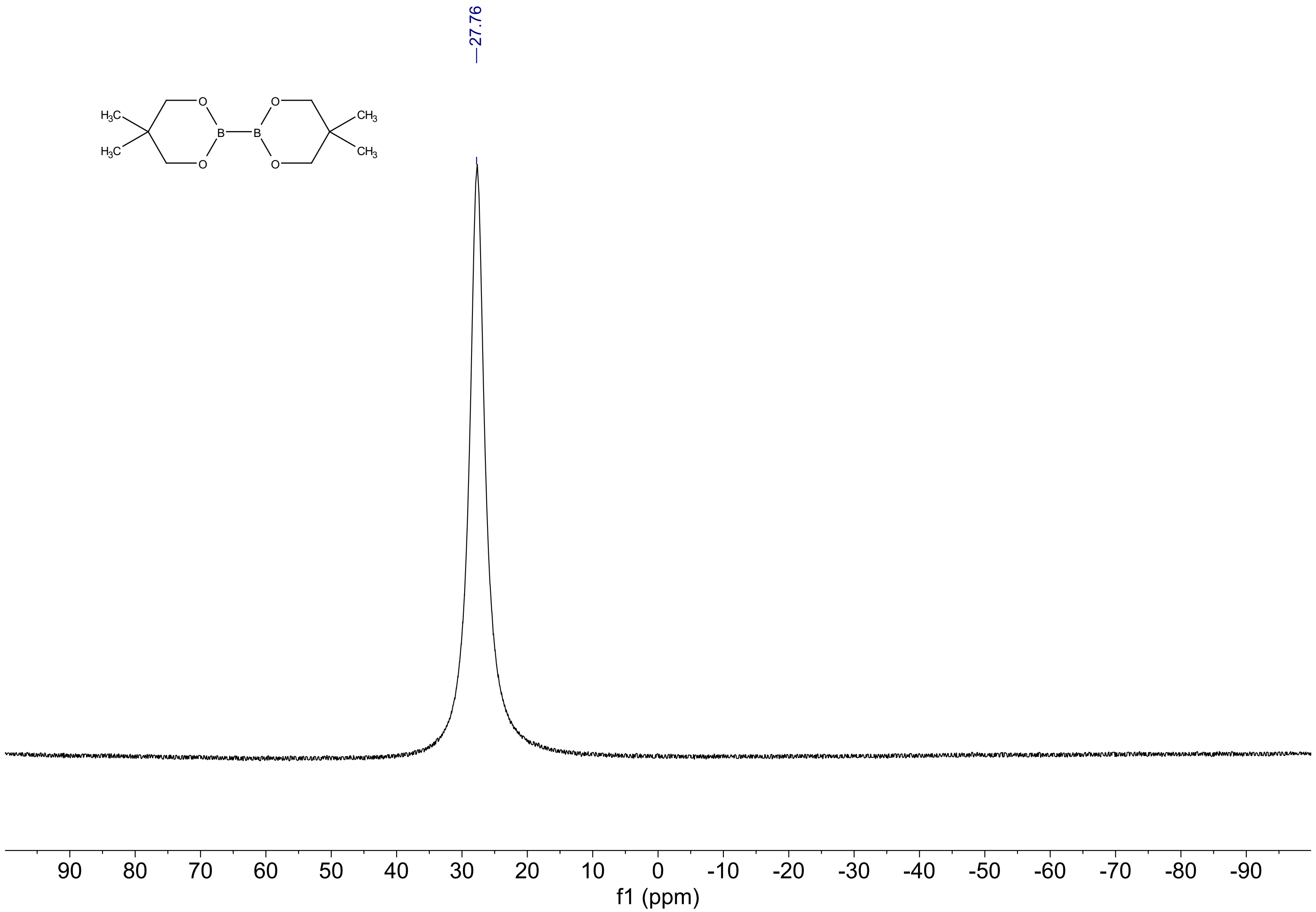

^{1}H NMR of *4,4,4',4',6,6'-Hexamethyl-2,2'-bi(1,3,2-dioxaborinane)* (12) ($CDCl_3$, 500 MHz)

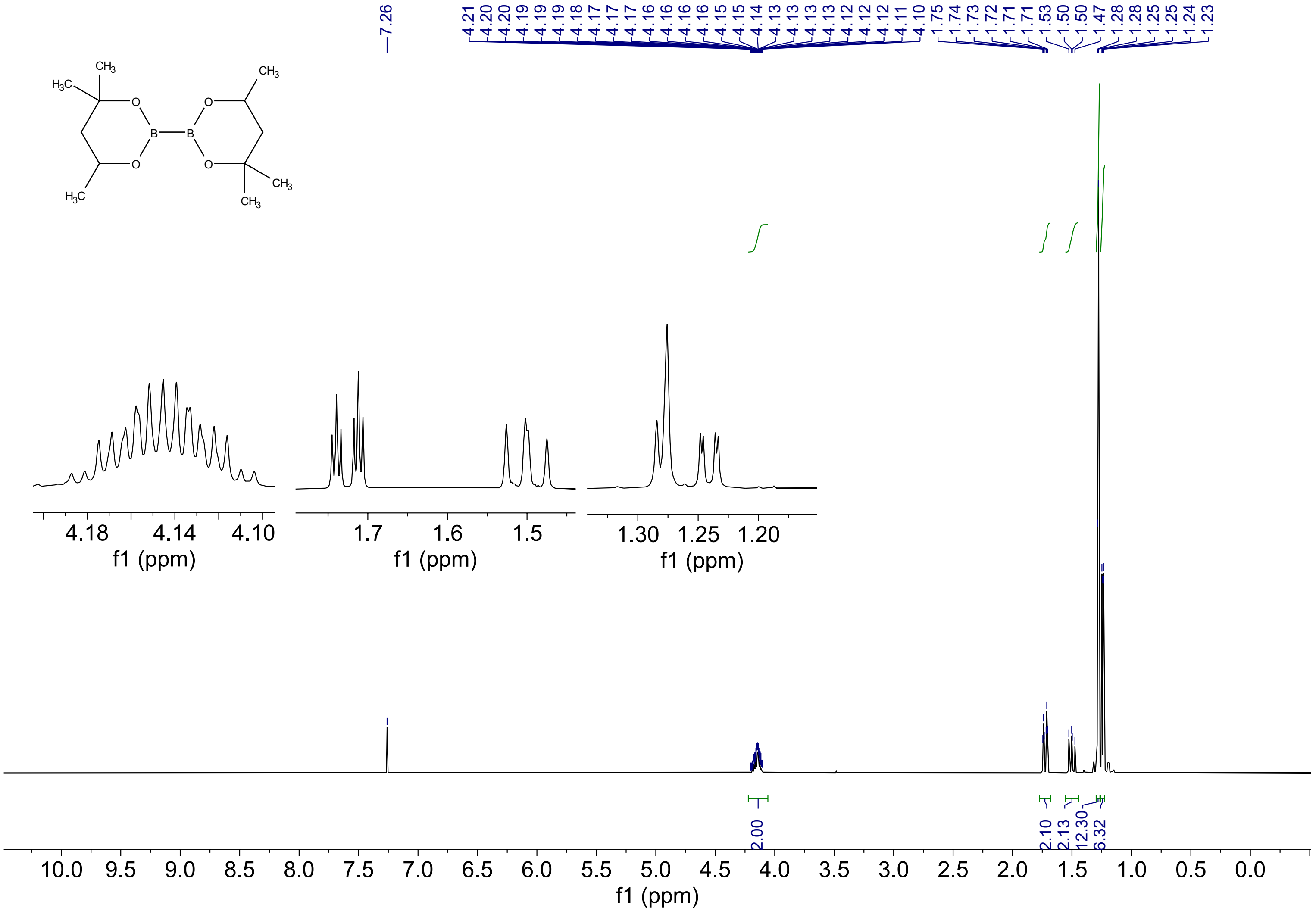

$^{13}C\{^{1}H\}$ NMR of *4,4,4',4',6,6'-Hexamethyl-2,2'-bi(1,3,2-dioxaborinane)* (12) ($CDCl_3$, 126 MHz)

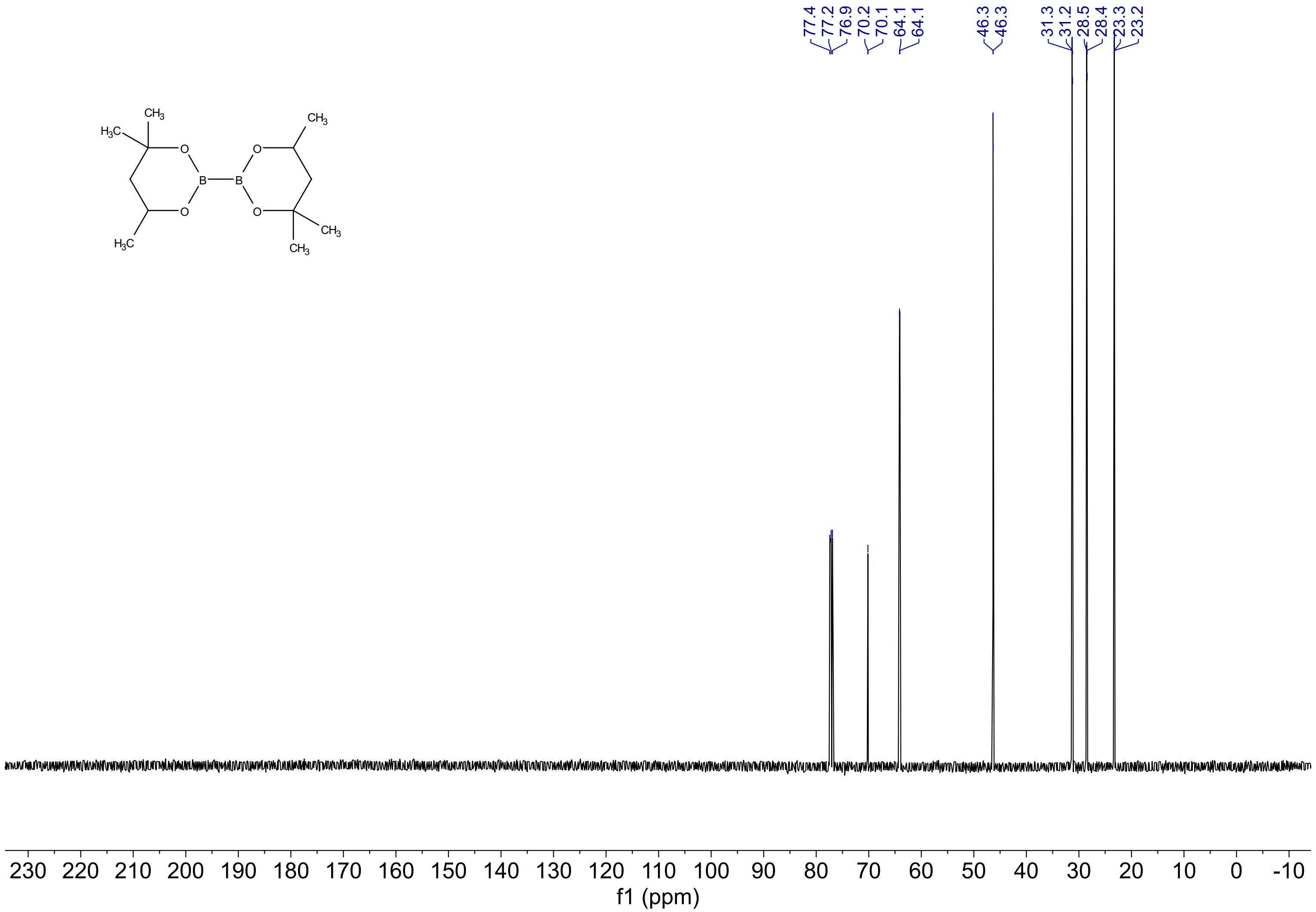

^{11}B NMR of *4,4,4',4',6,6'-Hexamethyl-2,2'-bi(1,3,2-dioxaborinane)* (12) ($CDCl_3$, 160 MHz)

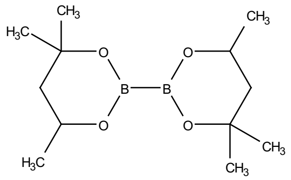

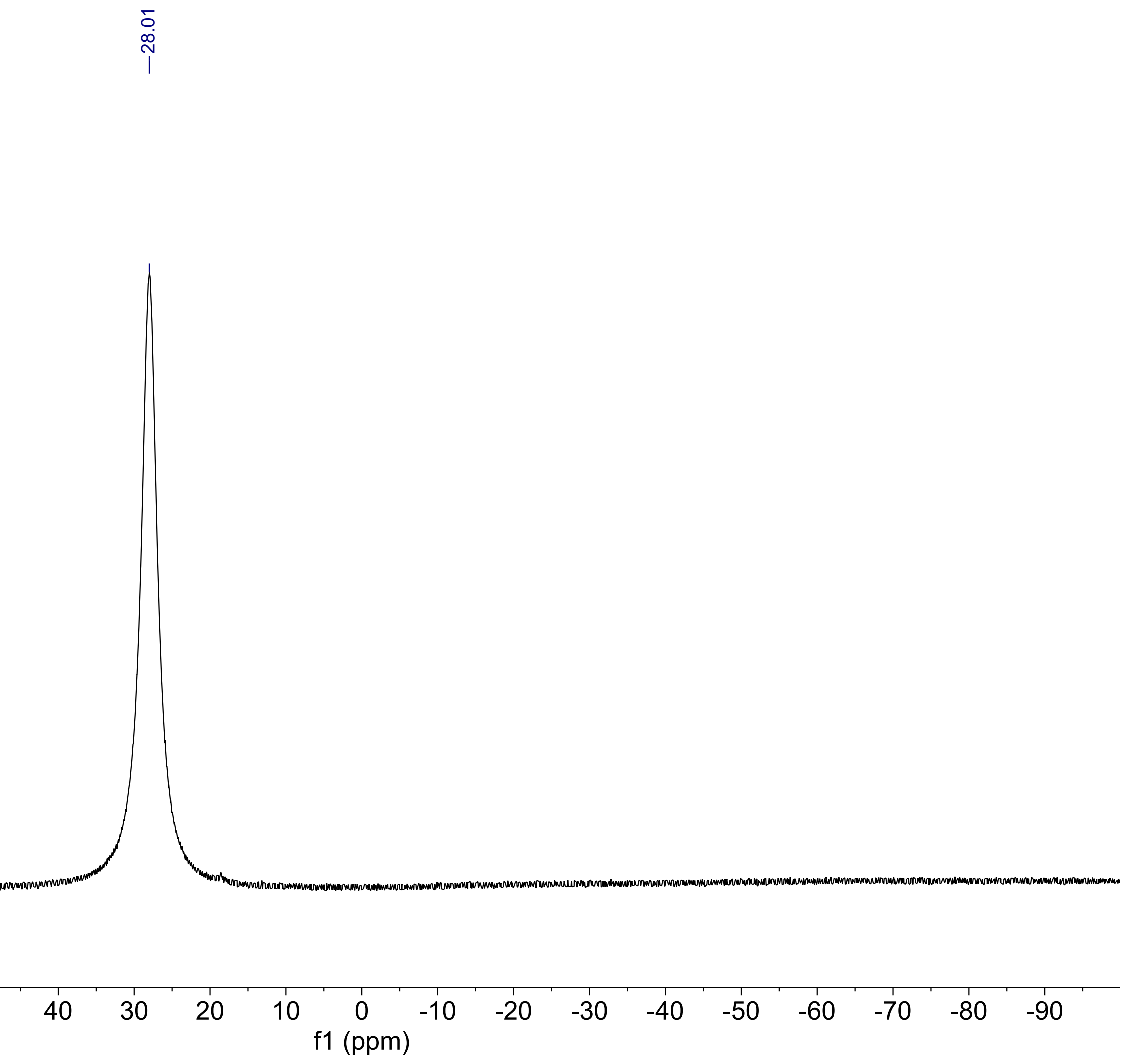

^{1}H NMR of *4,4,4',4'-tetramethyl-2,2'-bi(1,3,2)dioxaborinane* (13) ($CDCl_3$, 500 MHz)

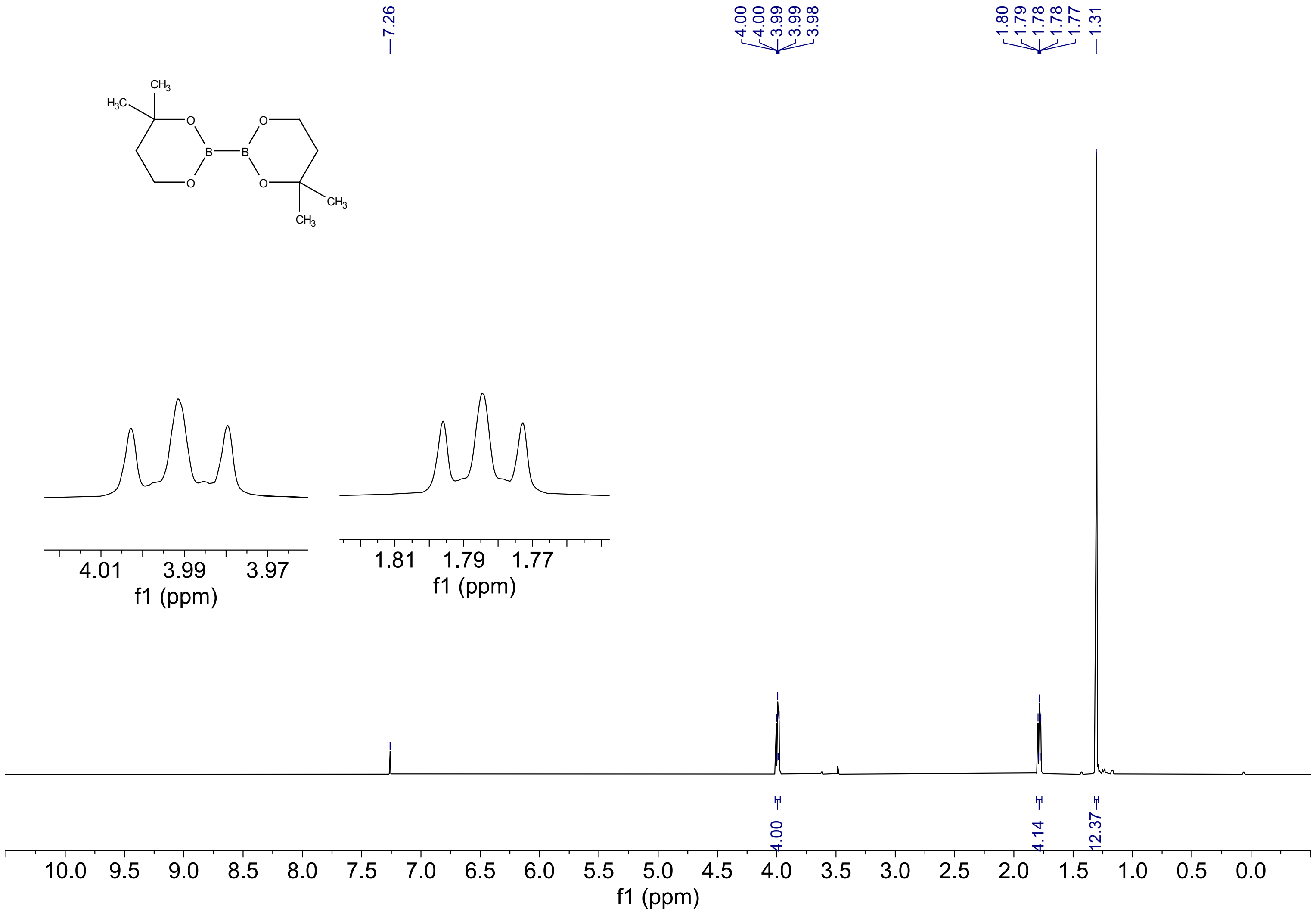

$^{13}C\{^{1}H\}$ NMR of *4,4,4',4'-tetramethyl-2,2'-bi(1,3,2)dioxaborinane* (13) ($CDCl_3$, 126 MHz)

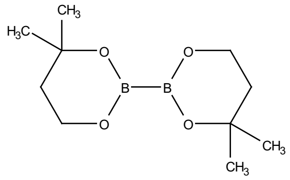

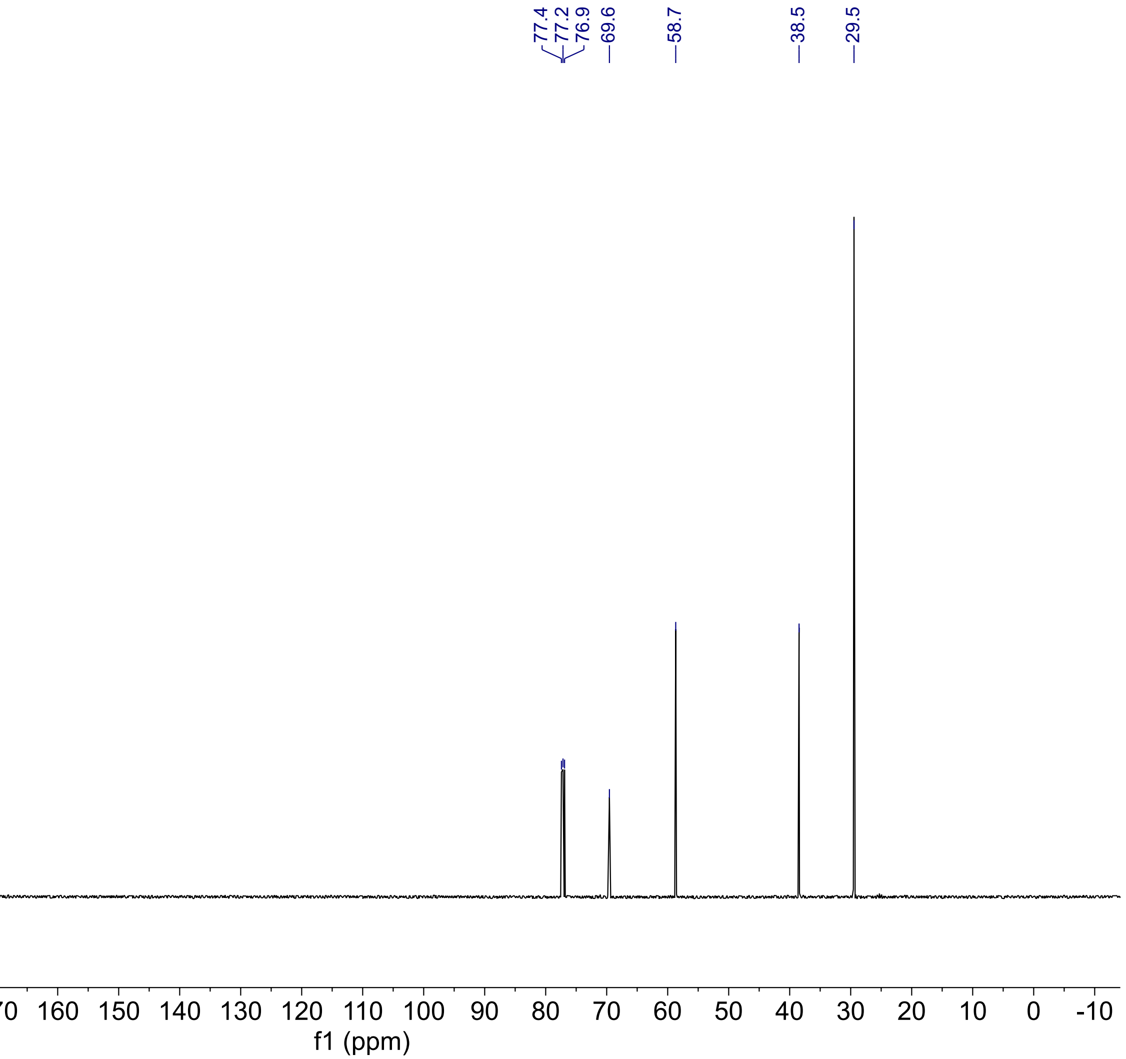

^{11}B NMR of *4,4,4',4'-tetramethyl-2,2'-bi(1,3,2)dioxaborinane* (13) ($CDCl_3$, 160 MHz)

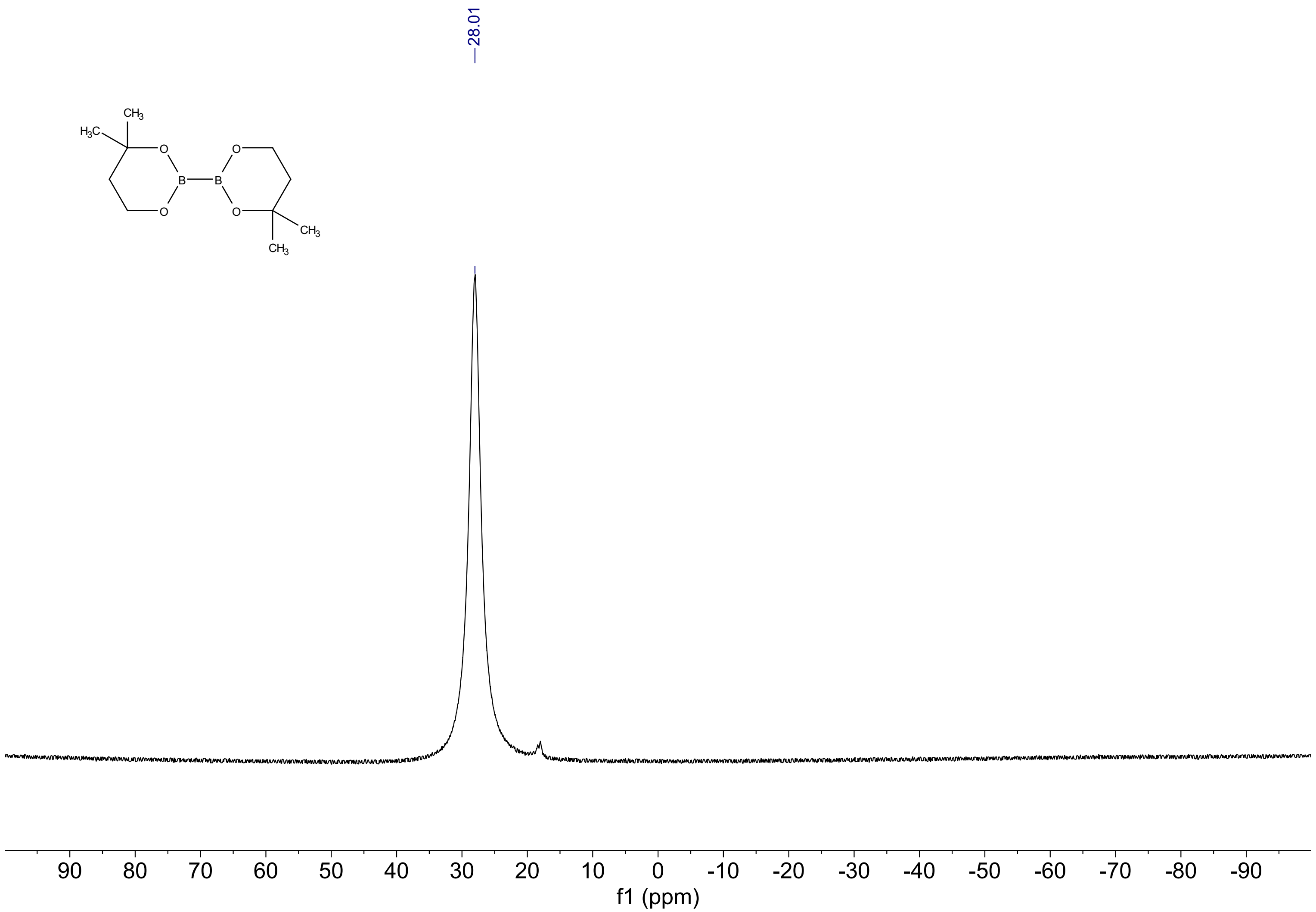

^{1}H NMR of *2,2'-Bibenzo[d][1,3,2]dioxaborole* (14) ($CDCl_3$, 500 MHz)

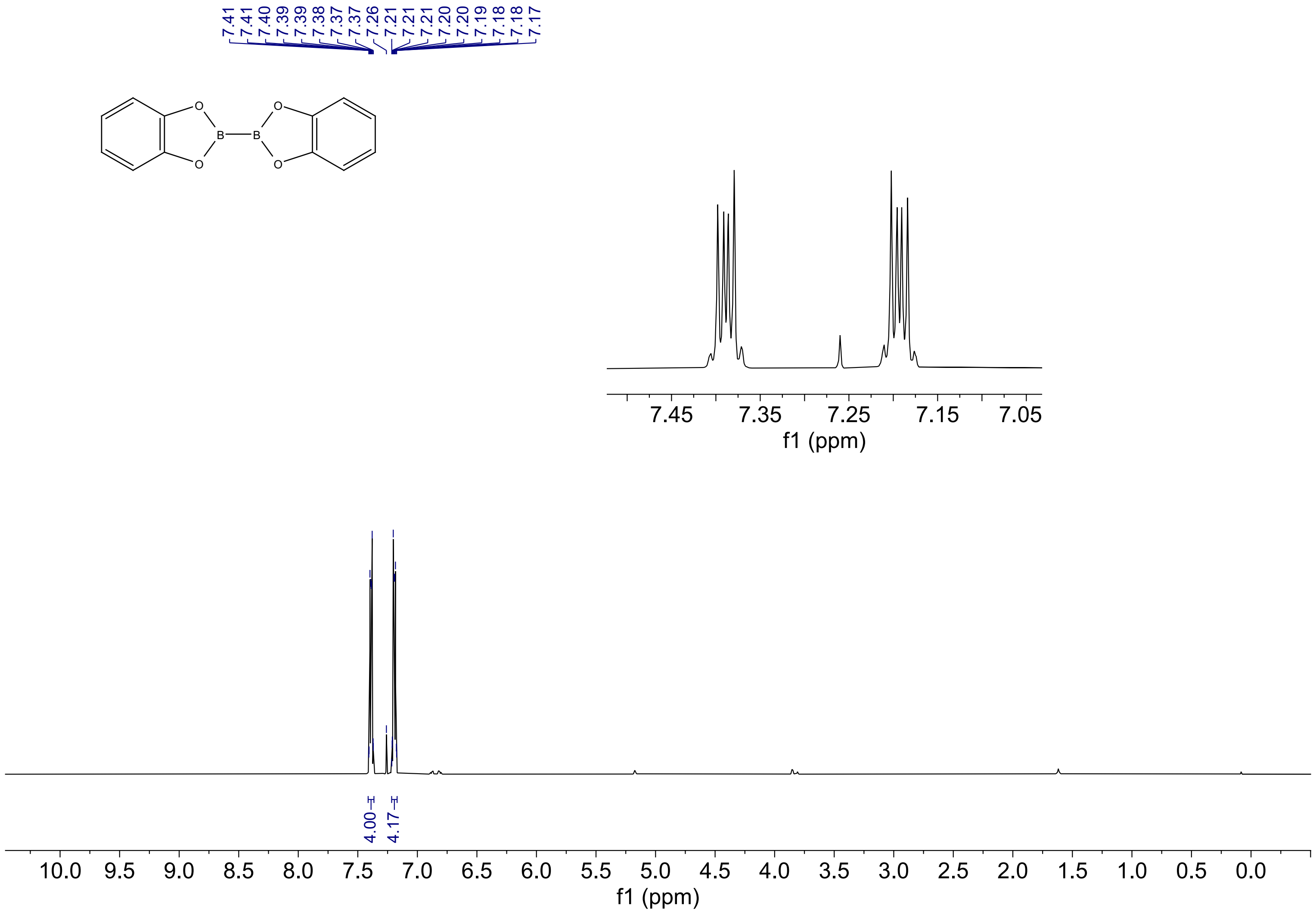

$^{13}C\{^{1}H\}$ NMR *of 2,2'-Bibenzo[d][1,3,2]dioxaborole* (14) ($CDCl_3$, 126 MHz)

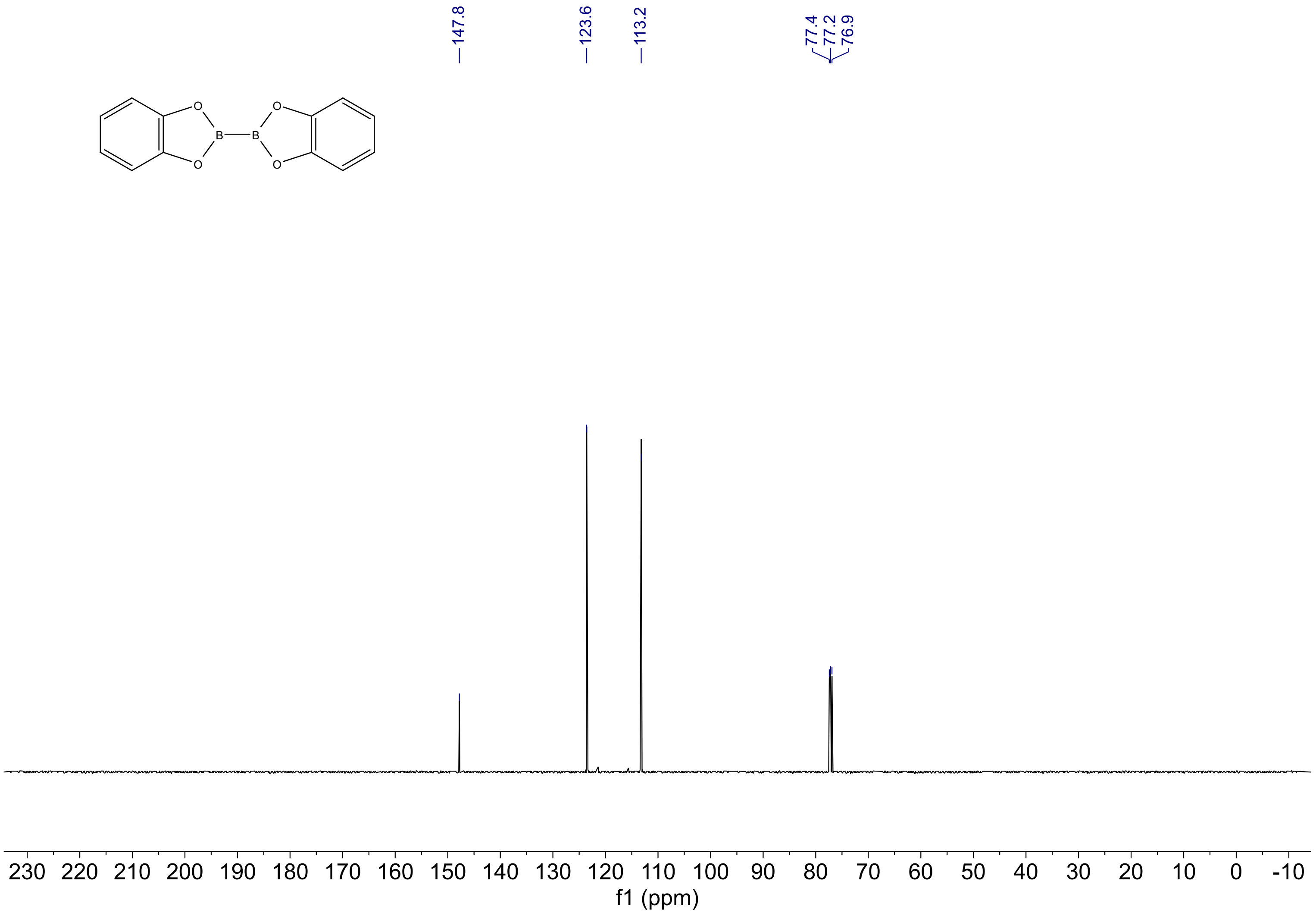

^{11}B NMR of *2,2'-Bibenzo[d][1,3,2]dioxaborole* (14) ($CDCl_3$, 160 MHz)

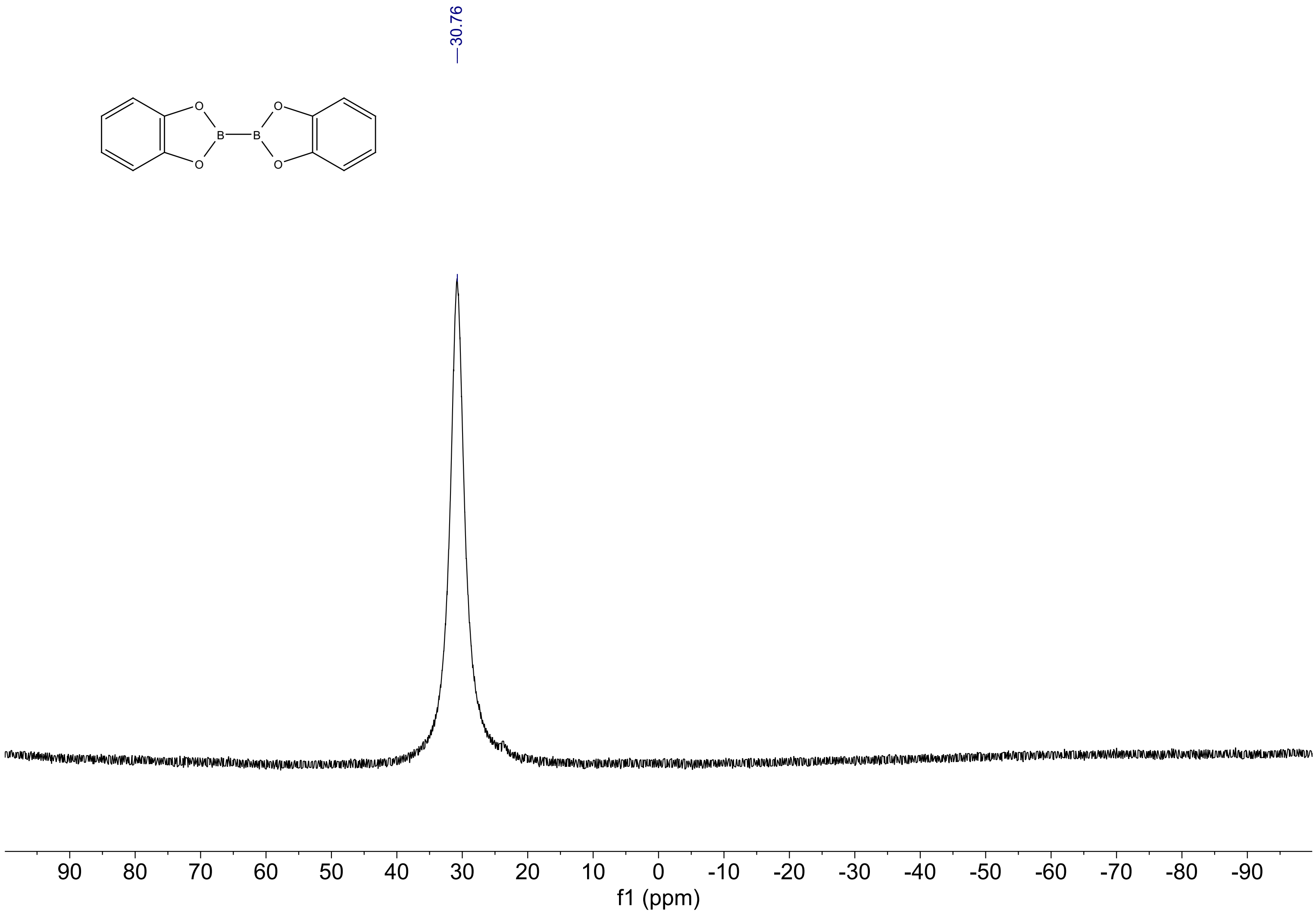

^{1}H NMR of *4H,4'H-2,2'-Bibenzo[d][1,3,2]dioxaborinine* (15) ($CDCl_3$, 500 MHz)

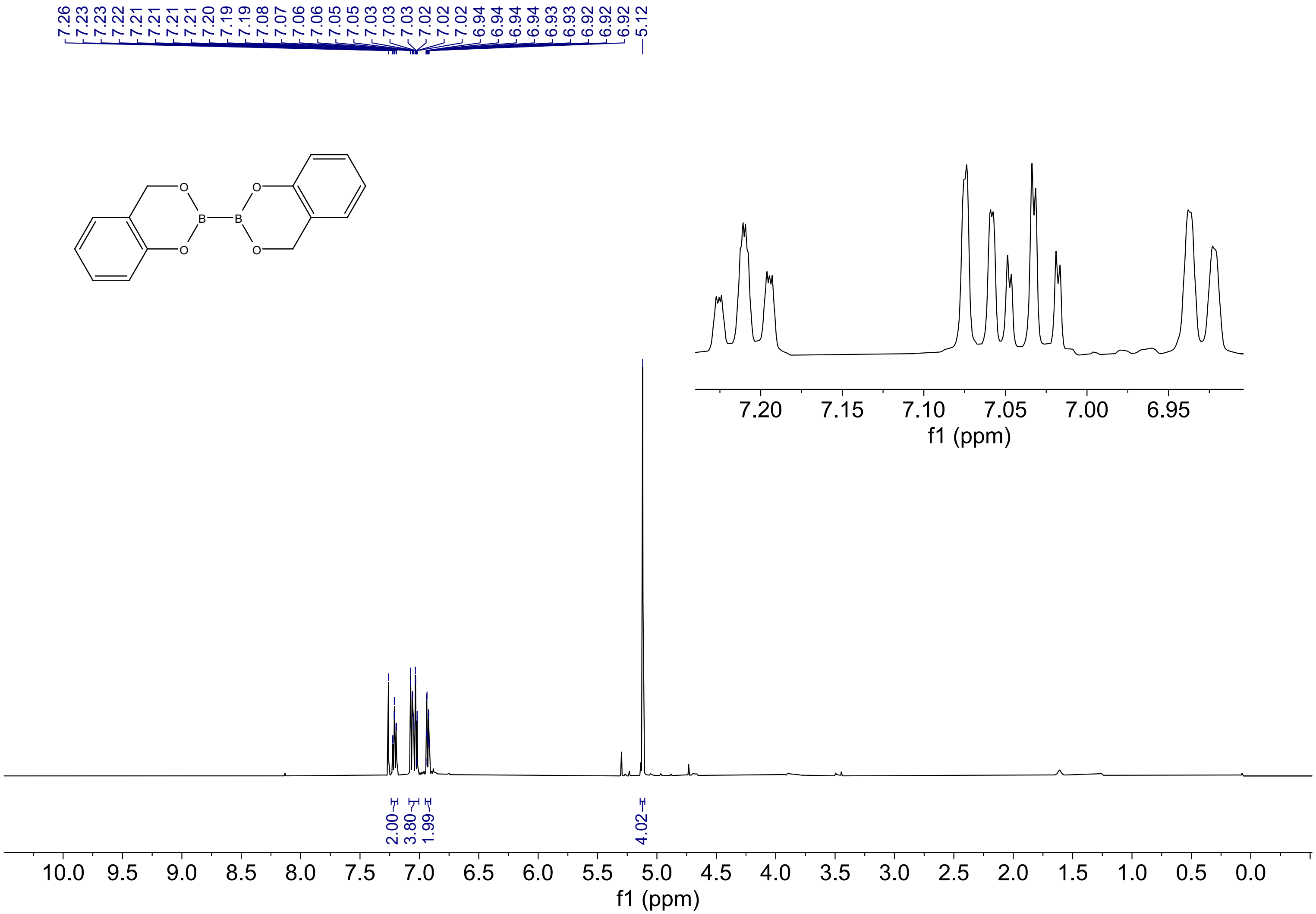

$^{13}C\{^{1}H\}$ NMR *of 4H,4'H-2,2'-Bibenzo[d][1,3,2]dioxaborinine* (15) ($CDCl_3$, 126 MHz)

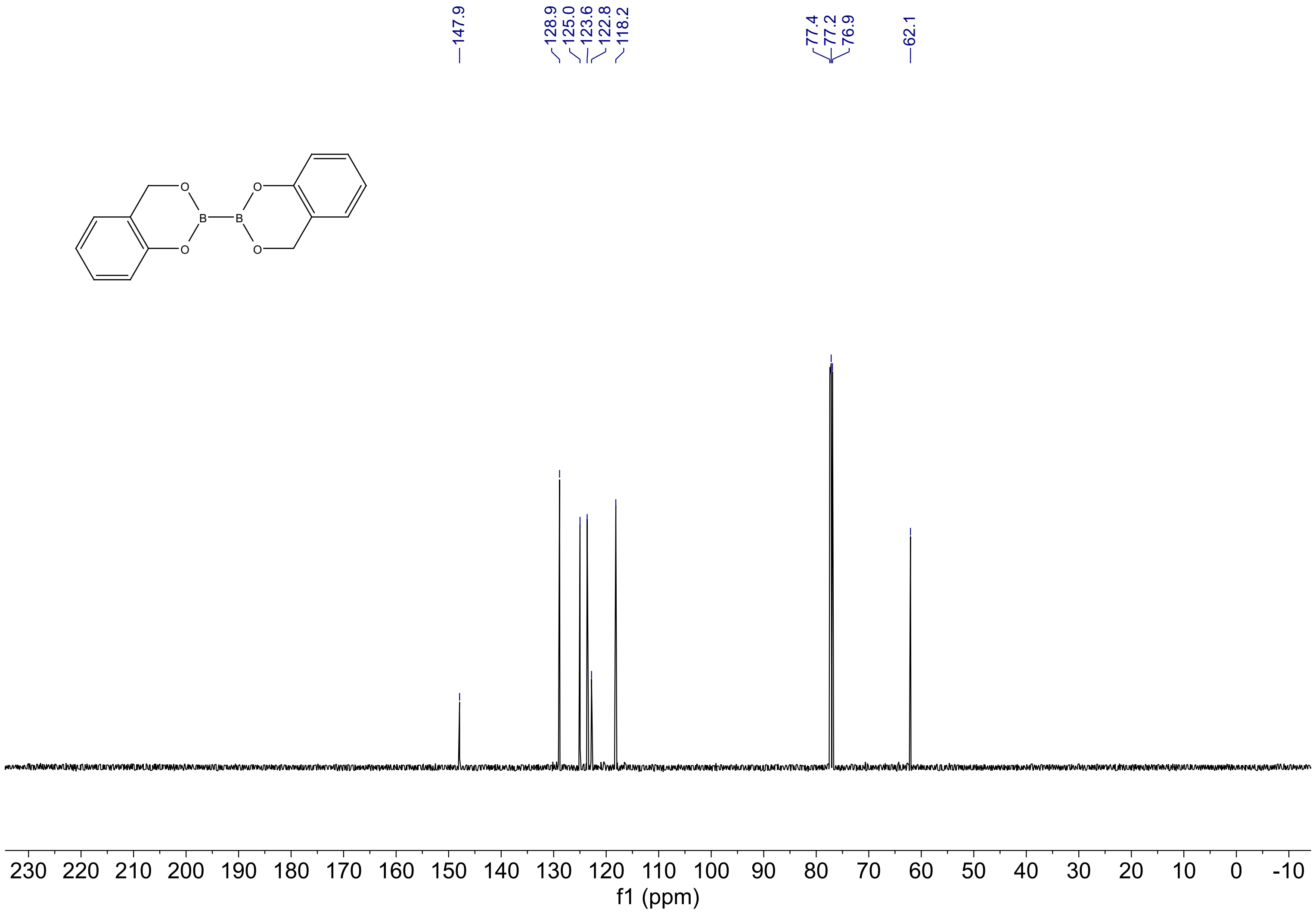

^{11}B NMR of *4H,4'H-2,2'-Bibenzo[d][1,3,2]dioxaborinine* (15) ($CDCl_3$, 160 MHz)

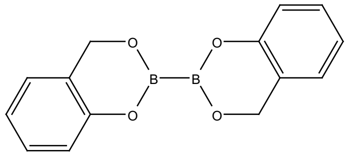

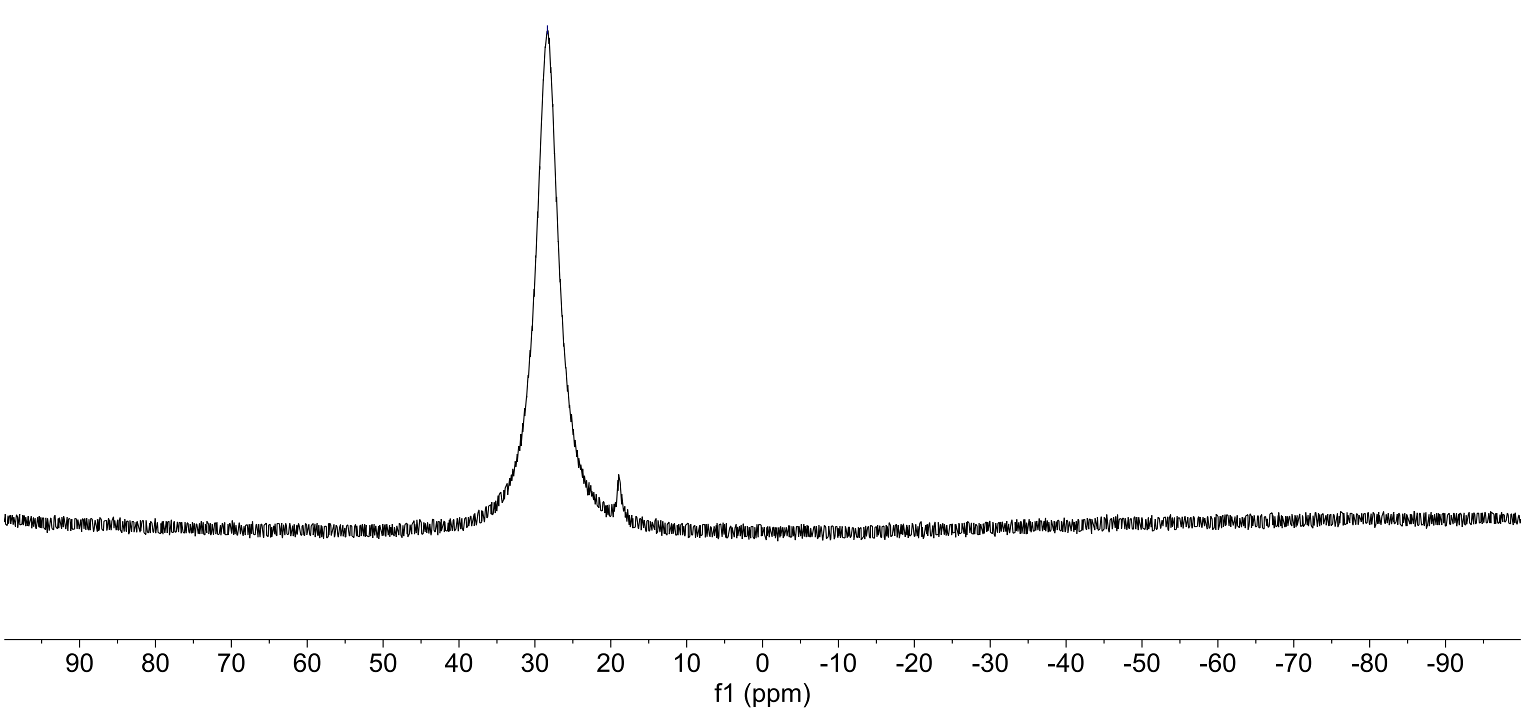

^{1}H NMR of *1,1',3,3'-tetrahydro-2,2'-bibenzo[d][1,3,2]diazaborole* (16) (($(CD_3)SO$, 500 MHz)

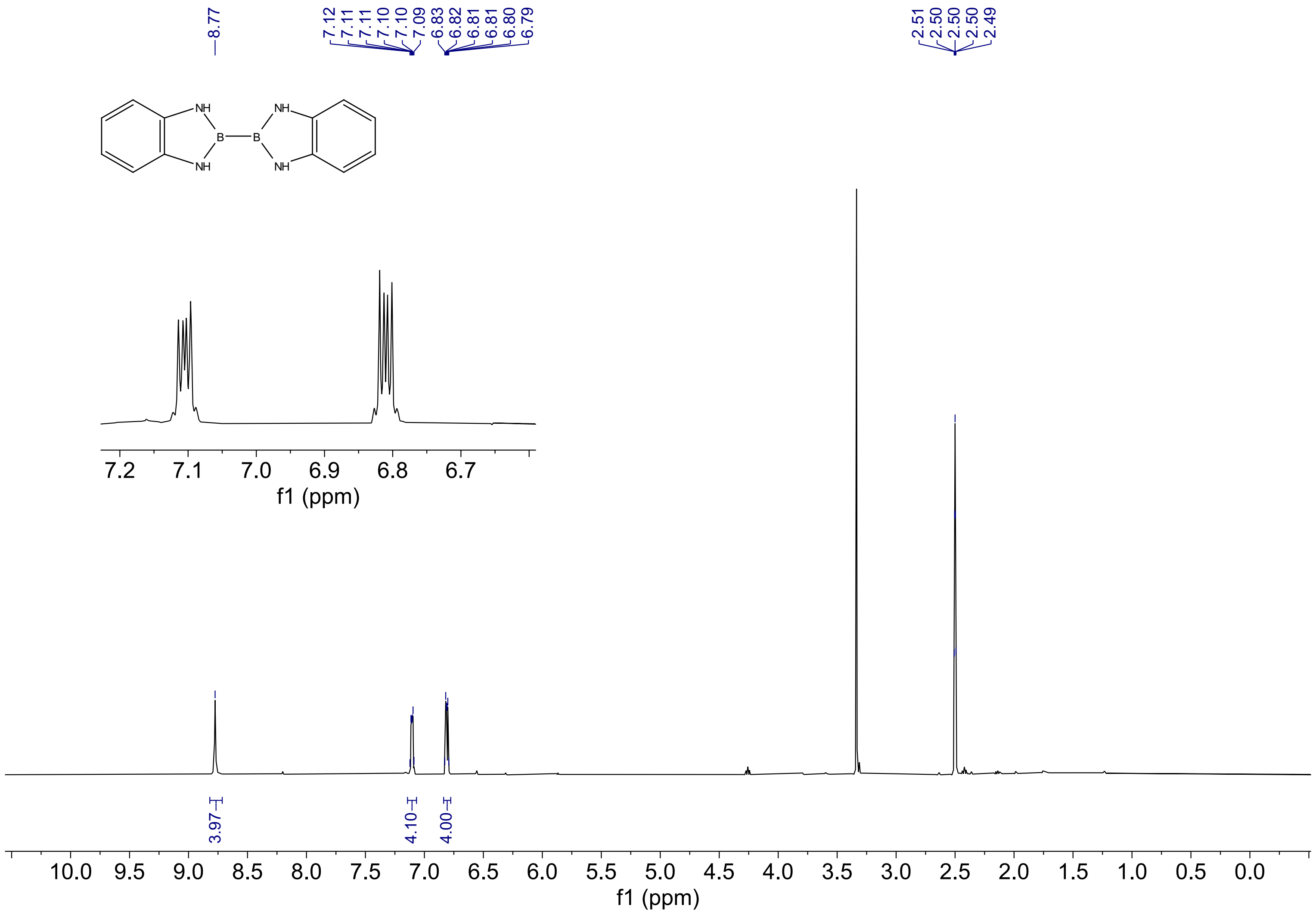

$^{13}C\{^{1}H\}$ NMR of *1,1',3,3'-tetrahydro-2,2'-bibenzo[d][1,3,2]diazaborole* (16) ($(CD_3)SO$, 126 MHz)

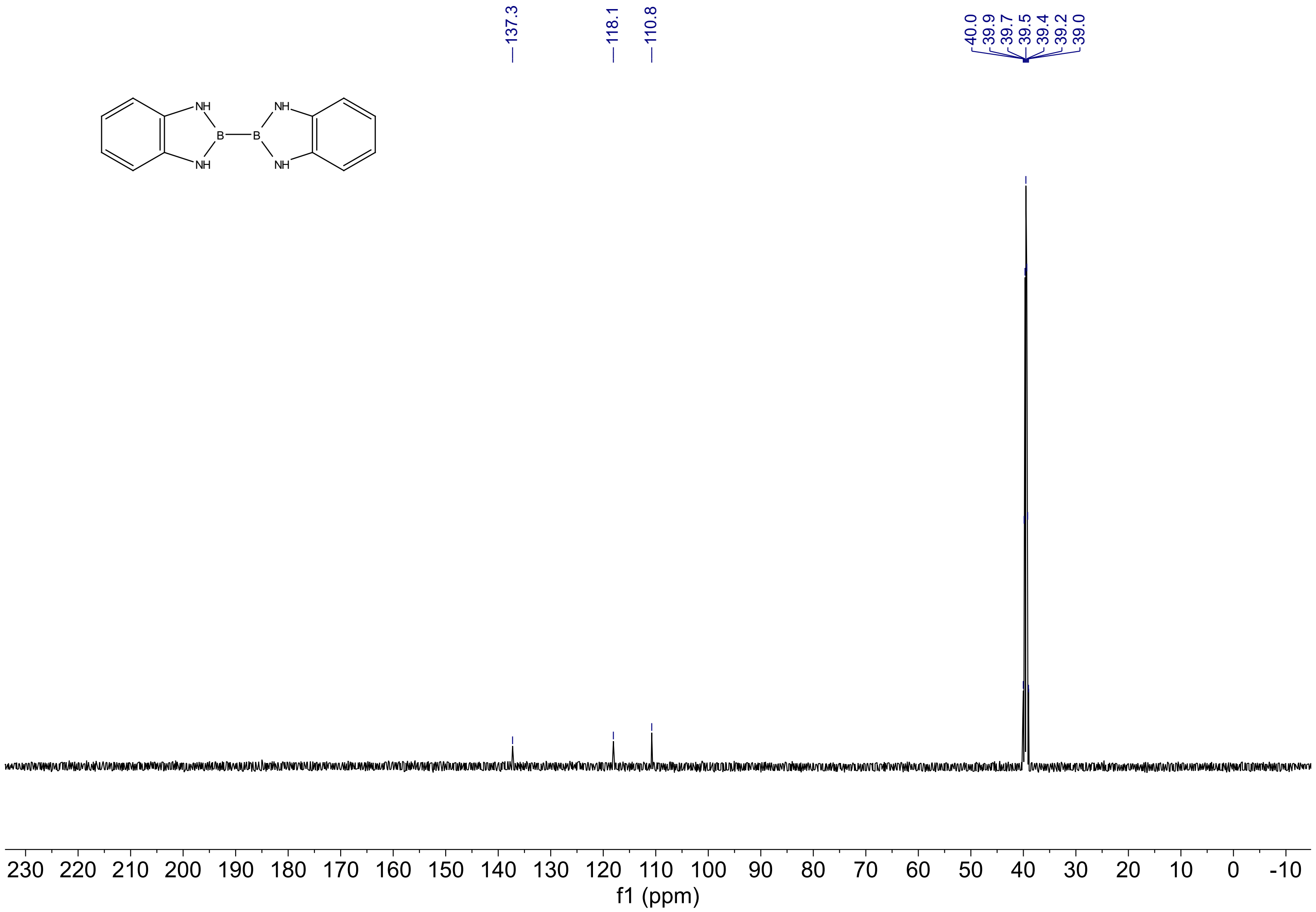

^{11}B NMR of *1,1',3,3'-tetrahydro-2,2'-bibenzo[d][1,3,2]diazaborole* (16) (C_3D_7NO, 160 MHz)

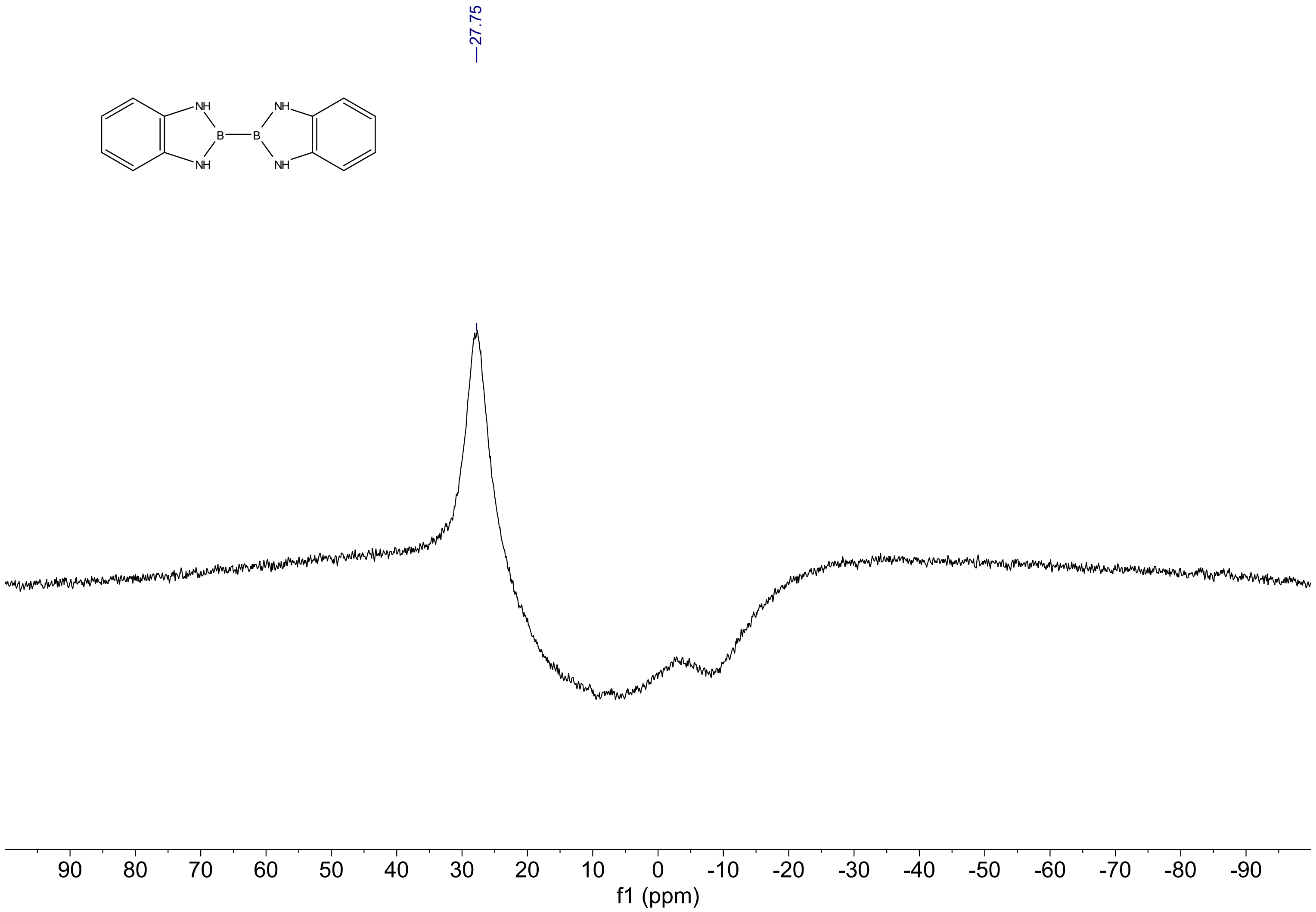

Supplement: Supplementary file 1 — jo3c02992_si_001.pdf [file jo3c02992_si_001.pdf]
